# Identification of a novel isoform of *Slc26a4* by single-cell RNA-sequencing of pendrin-expressing cells in the cochlea

**DOI:** 10.1007/s00439-026-02858-x

**Published:** 2026-07-16

**Authors:** Jin-Young Koh, Corentin Affortit, Kazuaki Homma, Satoe Takahashi, Jonathan M. Nizar, Paul T. Ranum, Rose Gogal, Eun-Mi Kim, Minkyung Kang, Diana L. Kolbe, Fengxiao Bu, Cody West, Donghong Wang, Amanda Odell, Amy Weaver, Jori Hendon, William D. Walls, Michael J. Schnieders, Richard J. H. Smith

**Affiliations:** 1 Roy J. Carver Department of Biomedical Engineering, College of Engineering, University of Iowa, Iowa City, IA USA; 2 Molecular Otolaryngology and Renal Research Laboratories, Carver College of Medicine, University of Iowa, Iowa City, IA USA; 3 Department of Otorhinolaryngology Head & Neck Surgery, School of Medicine, University of Maryland, Baltimore, MD USA; 4 Department of Otolaryngology – Head and Neck Surgery, Feinberg School of Medicine, Northwestern University, Chicago, IL USA; 5 Center for Mechanical Excitability, The University of Chicago, Chicago, IL USA; 6 The Hugh Knowles Center for Clinical and Basic Science in Hearing and Its Disorders, Northwestern University, Evanston, IL USA; 7 Department of Internal Medicine, Carver College of Medicine, University of Iowa, Iowa City, IA USA; 8 Raymond G. Perelman Center for Cellular and Molecular Therapeutics, The Children’s Hospital of Philadelphia Research Institute, Philadelphia, PA USA; 9 Department of Bio & Environmental Technology, College of Science and Convergence Technology, Seoul Women’s University, Seoul, Republic of Korea; 10 SillaJen Inc. , Seoul, Republic of Korea; 11 Institute of Rare Diseases, West China Hospital of Sichuan University, Chengdu, China; 12 Department of Otolaryngology, Head and Neck Surgery, Carver College of Medicine, University of Iowa, Iowa City, IA USA

**Keywords:** scRNA-seq, Novel isoforms, Cochlea, Hearing loss, *SLC26A4*, Pendred syndrome, NSEVA, DFNB4

## Abstract

Pathogenic variation of *SLC26A*4 gene causes both Pendred syndrome (PDS) and non-syndromic enlarged vestibular aqueduct (NSEVA/DFNB4), two autosomal recessive disorders. The former accounts for approximately 6% of human genetic hearing loss, making it the second most common form of syndromic deafness after Usher syndrome, while the latter is the most common radiological malformation associated with childhood sensorineural hearing loss (SNHL). Here, we used short- and long-read single-cell RNA sequencing (scRNA-seq) of pendrin-expressing cells in the murine cochlea to identify a novel short isoform of *Slc26a4*. We demonstrate that the short *Slc26a4* isoform is expressed in both the inner ear and kidney and investigate its interactions and functions. We also characterize the genotype-phenotype association for *SLC26A4*-related hearing loss in the context of these two isoforms. These results provide a new reference for molecular profiling of pendrin and offer novel insights into cell-type-specific splicing events and *SLC26A4*-related hearing loss.

**Supplementary Information:**

The online version contains supplementary material available at 10.1007/s00439-026-02858-x.

## Introduction

Genetic variation in *SLC26A4* is an important cause of hereditary hearing loss in humans. It is the primary genetic cause of both Pendred syndrome (PDS, OMIM #274600) and non-syndromic enlarged vestibular aqueduct (NSEVA/DFNB4, OMIM #600791) (Smith 1998–2020; Smith et al. 1993). The former accounts for ~ 6% of human genetic hearing loss, placing PDS as the second most common form of syndromic deafness after Usher syndrome, while the latter is the most common structural deformity of the bony labyrinth associated with childhood sensorineural hearing loss (SNHL) (Sloan-Heggen et al. 2016).

**Fig. 1:**
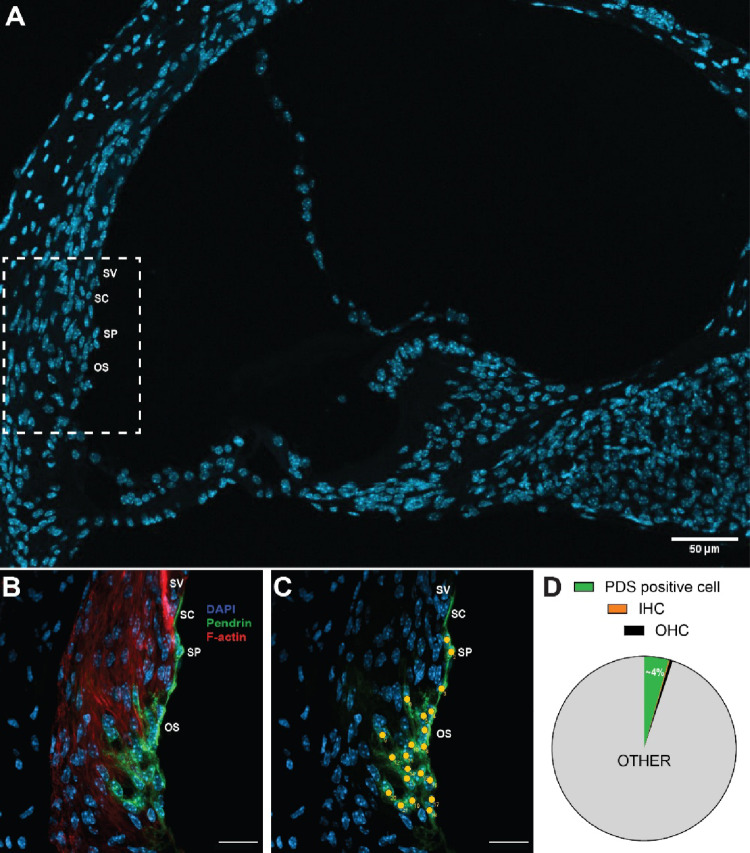
Estimate of pendrin-expressing cell in the membranous labyrinth of the adult mouse cochlea.** A** Representative confocal image of a mouse cochlea cross section stained with DAPI (blue) used to count total number of cells in the cochlear labyrinth. **B** High magnification showing pendrin (green), F-actin (red), nuclei (blue) in the stria vascularis (SV), spindle cells (SC), spiral prominence (SP), and outer sulcus (OS). Scale bar: 20 μm. **C** Pendrin positive cell count (yellow) Scale bar: 20 μm. **D** Pie chart showing the relative abundance of pendrin (PDS) positive cells compared to all membranous labyrinth cells. IHC, inner hair cells, OHC, outer hair cells

The *Slc26a4* encoded protein, pendrin, is a well described HCO_3_^−^/Cl^−^ antiporter that regulates endolymphatic pH homeostasis by HCO_3_^−^ secretion (Wangemann et al. 2007). In the inner ear, pendrin is expressed in the endolymphatic sac, in the utricle and in the spindle cells (SC) and root cells (RC) of the cochlear lateral wall (Wangemann et al. 2004; Koh et al. 2023; Cazals et al. 2015). These cells represent two of at least 37 different cell types in the murine membranous labyrinth (Jean et al. 2023), and in aggregate number only ~ 18,000 based on an estimated ~ 415,000 cells in the mature organ of Corti (Ehret and Frankenreiter 1977; Ranum et al. 2019) (Fig. 1). The scarcity of pendrin cells (PDCs) makes detailed study of these cell types challenging.

To address this challenge, we employed single-cell RNA sequencing (scRNA-seq) using a manual cell selection approach. Although manual cell selection is laborious and throughput is low, this approach does offer one important advantage compared to other techniques – it provides exceptionally high-quality cells for downstream analysis (Ranum et al. 2019; Koh et al. 2023; Liu et al. 2023a, b; Korrapati et al. 2019). By way of example, in earlier studies we used a manual technique to identify cell-type-specific splicing events and a large number of novel isoforms in genes associated with non-syndromic hearing loss that were not apparent with other single-cell RNA sequencing methods likely because of the transcriptional noise (Ranum et al. 2019; Liu et al. 2023a, b).

In this study, using manual cell selection coupled with short-read and long-read scRNA-seq, we identified a novel short *Slc26a4* isoform in PDCs (Fig. S1). We confirmed the expression of this novel isoform in the inner ear and kidney where canonical isoforms are expressed and studied its function *in vitro* with and without the canonical isoform of pendrin. In addition, we investigated genotype-phenotype associations in PDS and NSEVA/DFNB4 patients based on the predicted expression of this isoform.

## Results

### Quantification of gene expression using two single-cell RNA-seq libraries

We prepared two scRNA-seq libraries per isolated cell (Illumina, short read; ONT, long read) in parallel by dividing harvested full-length cDNA, as previously described (Byrne et al. 2017; Ranum et al. 2019) (Fig. S1). A mean of 2857 genes (range: 903–4367) across samples of the shared genes was identified by both sequencing technologies with an average transcript length of ~ 50,000 bp (Figs. S2).

### Illumina short-read and Nanopore long-read scRNA-seq uncover a novel transcription start site in *Slc26a4*

**Fig. 2:**
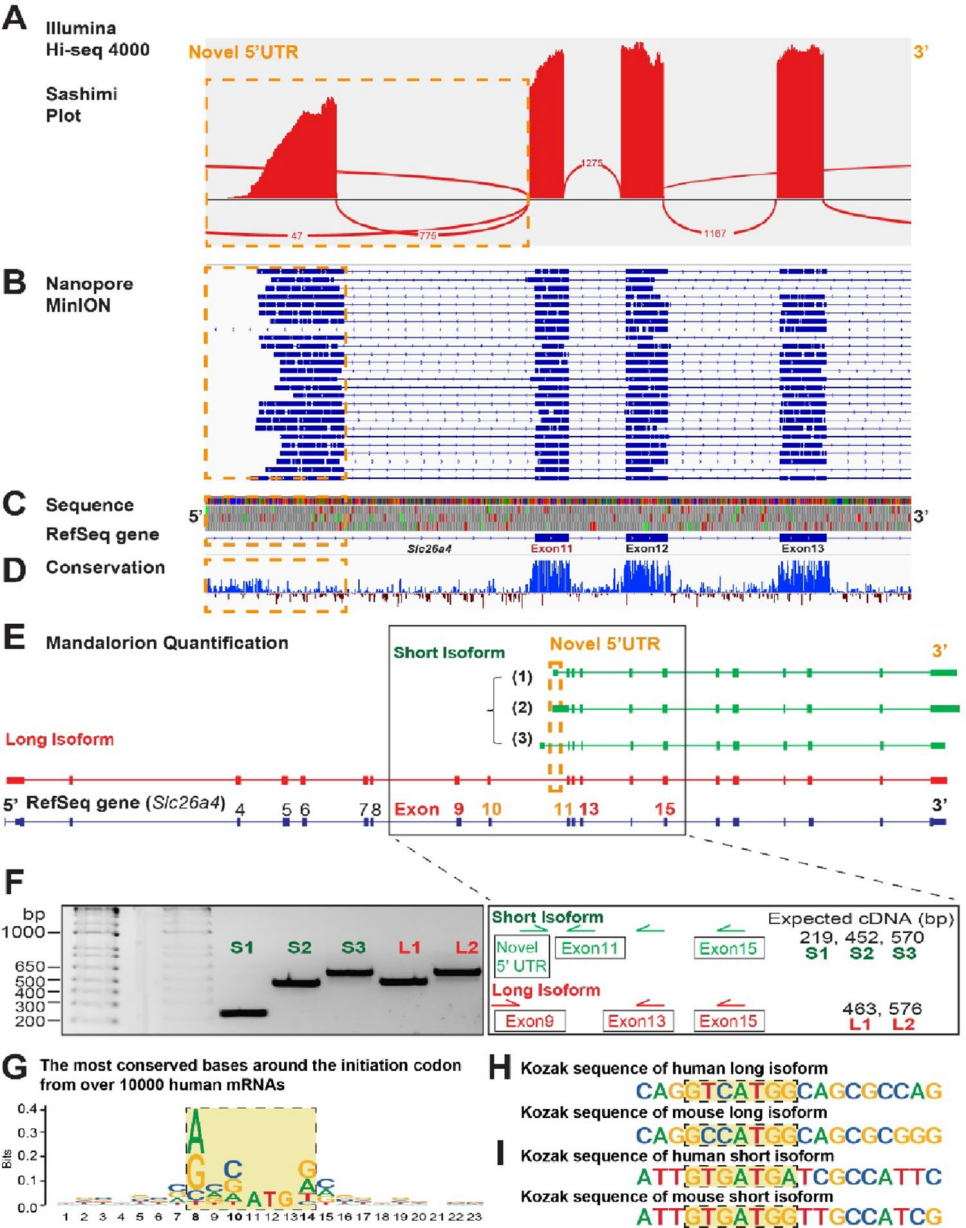
Novel isoform of ***Slc26a4*** and validation of the novel short isoform.** A-E** The novel 5’UTR preceding exon 11 is highlighted in the dashed yellow rectangles. **(A)** The sashimi plot of IGV depicts the novel exon and number of reads (775). **B** Long-read scRNA-seq shows that the novel exon is the first exon of the short isoform **C** A 5’-UTR sequence without the presence of a methionine start codon is shown in the dashed rectangle region. The novel exon is unannotated in RefSeq. The methionine start codon for the short isoform is aa residue 432 of the long isoform. **D** The phyloP placental mammal basewise conservation from 60 species shows a small peak before the novel transcription start site. **E** Isoform consensus sequence alignments generated by Mandalorion and aligned to the mm10 genome. The novel short (1–3) and known long (4) isoforms of *Slc26a4* are shown above the RefSeq annotation. **F** Validation of mRNA expression using cochlear lateral wall (30 ng) by RT-PCR. The electrophoresis gel image shows the expression of the short isoform using a forward primer based on the novel UTR and three different reverse primers. Expression of the long isoform uses a forward primer in exon 9 and two different reverse primers. The nucleotide ladder is on the left (200 to 1000 bp). The novel 5’UTR for the short isoform is found between exons 10 and 11. **G** The sequence logo illustrates the highly conserved bases adjacent to the initiation codon (ATG). The size of the letters is a clear indicator of the frequency with which these bases are incorporated, with larger letters signifying greater prevalence. It’s particularly striking to observe the prominence of A and G at the 8th position (−3, Kozak position) and the strong representation of G at the 14th position (+ 4, Kozak position). **H-I** Kozak consensus sequence of the long (**H**) and short (**I**) isoforms in Homo sapiens and Mus musculus

Results were remarkable for a novel 5’ untranslated region (UTR) in *Slc26a4* identified by both short- and long-read scRNA-sequencing, which lies between exons 10 and 11 and for which there is no RefSeq (Pruitt et al. 2014) annotation (Fig. 2A-C). Small peaks from the phyloP placental mammal base-wise conservation were present that align directly upstream of the novel transcription start site TSS (Fig. 2D). Translation of mRNAs originating from this novel 5’ UTR uses an open reading frame (ORF) starting at a methionine codon in exon 11. We used the Mandalorion pipeline (Byrne et al. 2017) to quantify isoform diversity and detected two different isoforms of *Slc26a4*: the canonical long isoform and the short isoform (Figs. 2E, S3).Protein sequence alignment of pendrin short isoforms reveled high homology (86% identity and 94% similarity) between human and mouse. To validate the transcription of this short isoform, we documented mRNA expression by targeting the novel 5’UTR in mouse cochlea (Fig. 2F) and in human thyroid and kidney tissues (Fig. S4).

The Kozak consensus sequence is a specific sequence of nucleotides found in eukaryotic mRNA that plays a critical role in the initiation of translation. This sequence surrounds the start codon (AUG) and enhances the recognition of mRNA by the ribosome. In most eukaryotic mRNA transcripts, adenine (A) and guanine (G) are present in the three nucleotides upstream and immediately after the start codon. We confirmed the presence of this Kozak consensus sequence in both long (exon 2 of the canonical transcript) and short (exon 11 of the canonical transcript) pendrin isoforms in humans and mice (Figs. 2G-I).

### Structure and expression of the long and short pendrin isoforms

**Fig. 3:**
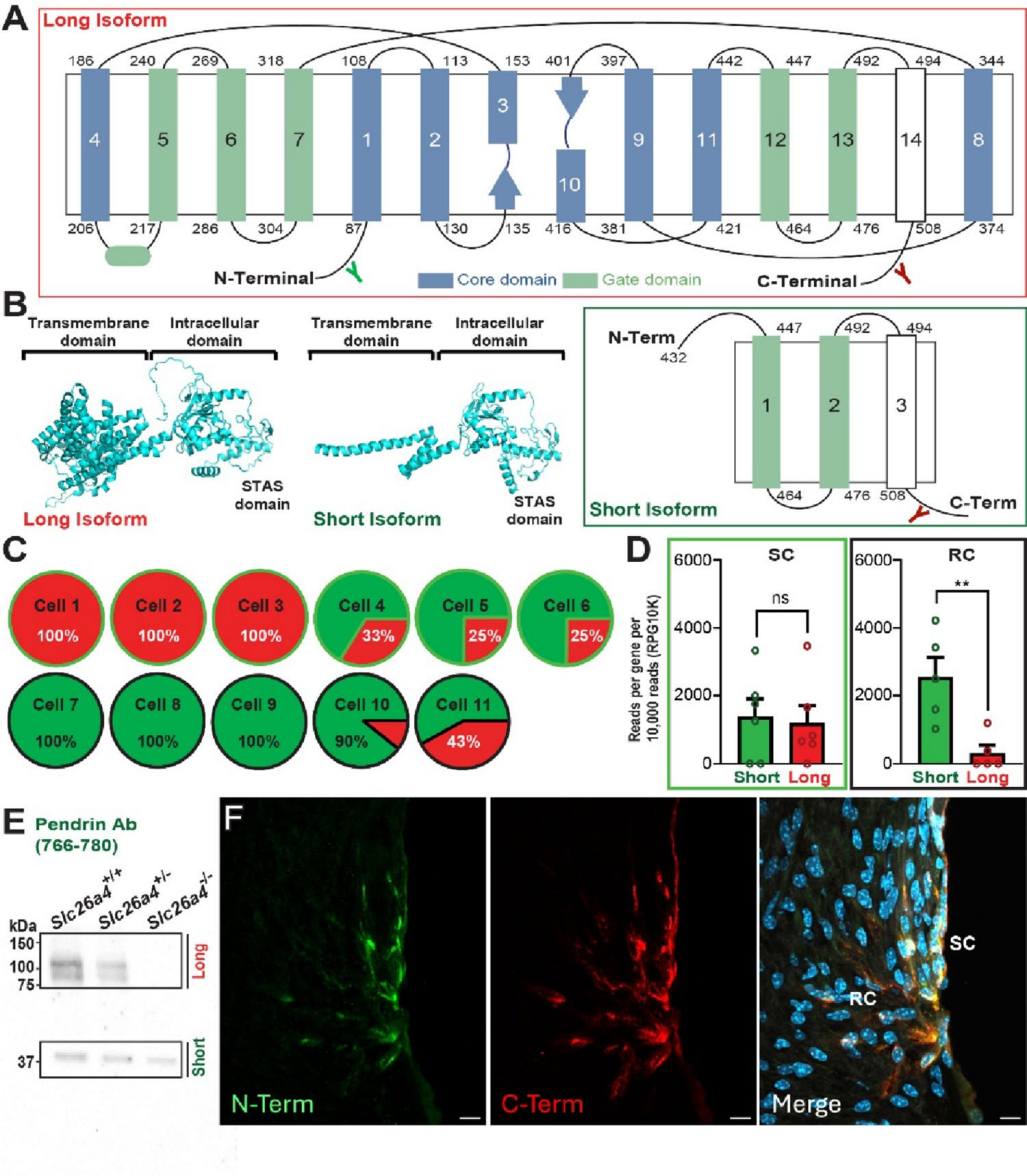
Expression of the long and short pendrin isoforms.** A** Schematic representation of the 14 transmembrane (TM) segment model of pendrin adapted from (Bassot et al. 2017) and the topology of the short isoform. The short isoform starts at amino acid (aa) 432 of the long isoform. MEMSAT-SVM predicts 3 TMs, which are identical to TMs 12–14 of the long isoform. The positions of the peptides used for generating the N-term (33–47) and C-term 766–780 antibodies are represented in green and red respectively. **B** Predicted AlphaFold2.0 model of the long (left) and short (right) isoforms of pendrin. The STAS domain is highlighted in both isoform complex models. Note: In the short isoform most of the transmembrane domains are missing. **C** Pie chart showing relative expression of the long (red) and short (green) isoforms at a single cell level in 11 PDCs. Note: Three of six spindle cells (green circumference) express only the long isoform, and three of five root cells (black circumference) express only the short isoform. **D** Histogram showing the expression levels per 10,000 reads (RPG10K) of the short and long isoforms of *Slc26a4* in spindle cells (SC) and root cells (RC). Data are expressed as mean ± SEM (*n* = 6 and *n* = 5 for SC and RC, respectively). Students’ t-tests were performed. ns: not significant, **: *p* < 0.05. **E** Representative western blot images showing cochlear expression of the long and short isoforms. Anti-pendrin antibodies (766–780) detect both the long and short isoforms of pendrin. *Slc26a4*^+/+^ (WT), *Slc26a4*^+/−^ (HET), and *Slc26a4*^−/−^ (PDSKO) mice aged 1 month are used as negative and positive expression controls. **F** Representative confocal images of the spiral ligament immunostained for pendrin N-term (long isoform) Green, pendrin C-term (both isoforms) red, phalloidin (grey) and nuclei (blue). **Note**: In the SC, the N-term and C-term labeling intensities are comparable and co-localize perfectly. In RC, higher C-term labeling intensity is observed suggesting a cell-type-specific isoforms expression. Scale bar 10 μm SC: spindle cells, RC: root cells

The long isoform of *Slc26a4* has 21 exons and encodes a 780-amino-acid (aa) (Everett et al. 1997). The structure of this isoform has recently been resolved using cryogenic electron microscopy (Liu et al. 2022; Wang et al. 2024) showing its transmembrane, intracellular, and STAS (Sulfate Transporter and Anti-Sigma factor antagonist) domains (Fig. 3B). In comparison, the short isoform, has 11 exons (Fig. 2E) and encodes a protein of 349 aa that starts at position 432 of the long isoform (Fig. 3A). Using MEMSAT-SVM (Nugent and Jones 2009), we predicted that the short isoform has three transmembrane (TM) domains, with TM3 being pore-lining (Fig. 3A-B)..

We used scRNA-seq to quantify mRNA expression of both isoforms by cell type (Fig. 3C). Based on previously described cell-specific genetic markers (Koh et al. 2023; Gu et al. 2020), we classified 11 PDCs as either spindle cells (*Anxa1*) and root cells (*Epyc*). Three of six spindle cells (SC, green circumference in Fig. 3C, top) expressed only the long isoform, while three of five root cells (RC, black circumference in Fig. 3C, bottom) expressed only the short isoform. Expression levels of the short and long isoforms of *Slc26a4* were not significantly different in SCs, but the short isoform expression was higher in RCs (Fig. 3D). These results suggest that there may be cell-specific differences in isoform expression between RC and SC. We confirmed protein expression of both, long and short isoforms (110 kDa and 39 kDa), using western blot method in the cochlea (Fig. 3E) and kidney (Fig. S5). In control animals (*Slc26a4*^*+/+*^), both isoform are expressed, by comparison, in *Slc26a4*^*−/−*^ mice, expression of the short isoform was reduced and the long isoform was undetectable, an expected finding since translation of the short isoform is initiated in exon 11 and the KO line was generated by targeted deletion of exon 8 (Everett et al. 2001) (Fig. 3E).

Cell-type cochlear expression and localization of the short and long isoforms were assessed in cochlear sections using N-terminal and C-terminal specific antibodies. In the SC, labeling intensity of the N-terminal and C-terminal antibodies was similar, while in the RC, C-terminal antibody staining was higher (Fig. 3F), indicating more abundant short isoform expression in these cells. These results suggest a cell-type-specific isoforms expression between the SC and RC.

### Interaction between long and short pendrin isoforms

**Fig. 4:**
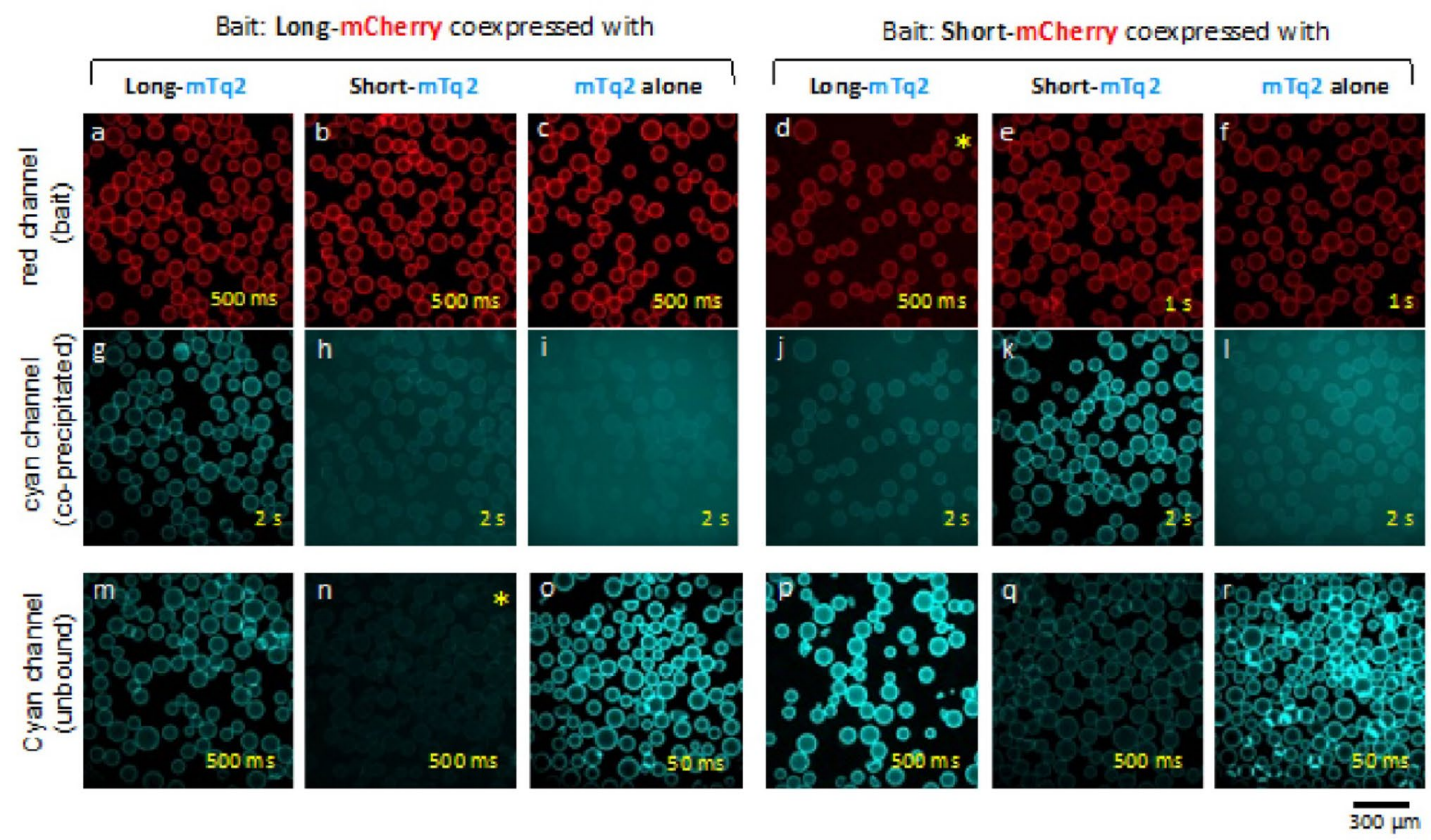
Interaction between long and short pendrin isoforms. mCherry-tagged long or short human pendrin construct (bait) was co-expressed with each of mTq2-tagged human pendrin construct (prey) in HEK293T cells as indicated, solubilized in a mild detergent-containing buffer, and pulled down with RFP-selector. Red fluorescence-positive beads (top panels a-f) indicate successful pull-down of the mCherry-tagged long or short human pendrin construct. Detection of cyan fluorescence indicates binding between bait and prey (middle panels g-l). The unbound fractions (flow-through) were incubated and pulled down with GFP-selector to collect unbound mTq2 (bottom panels m-r). Numbers in yellow indicate the camera exposure time. “*” indicates images that are enhanced more than the others for visibility. Scale bar indicates 300 μm

Recent single-particle cryo-EM studies have shown that pendrin forms a homodimer (Liu et al. 2023a, b; Wang et al. 2024). The short pendrin isoform lacks the entire N-terminal cytosolic and most transmembrane regions but retains the C-terminal region, which is important for dimerization (Fig. 3A), pointing to the possibility that the short isoform may form a homodimer or a heterodimer with the long isoform. To explore this possibility, we established stable cell lines co-expressing mCherry-tagged long or short human pendrin isoform and mTurquoise2 (mTq2)-tagged long or short human pendrin isoforms in four different combinations, i.e., Long-mCherry/Long-mTq2, Long-mCherry/Short-mTq2, Short-mCherry/Long-mTq2, and Short-mCherry/Short-mTq2 (Fig. S8). Cells co-expressing mCherry-tagged long or short human pendrin isoform and mTq2 were also established as negative controls. After cells were lysed, detergent-solubilized fractions were incubated with anti-mCherry-conjugated beads, RFP-selector. As expected, both long and short mCherry-tagged pendrin isoforms were successfully captured by RFP-selector (Fig. 4, top panels a-f). The binding of mTq2-tagged pendrin constructs to mCherry-tagged pendrin constructs was examined by observing cyan fluorescence on RFP-selector (Fig. 4, middle panels g-l). The presence of mTq2 or mTq2-tagged pendrin constructs in the RFP-selector-unbound fractions was examined using anti-mTq2-conjugated beads, GFP-selector (Fig. 4, bottom panels m-r). The expression of mTq2 (without tagging) was very high (notice the shortest camera exposure time for ‘mTq2 alone’ in panels o and r). The weak cyan fluorescence on RFP-selector (Fig. 4, panels i and l) was probably due to nonspecific mTq2 binding augmented by the high mTq2 expression. The high background fluorescence in these images suggests a fast rate of mTq2 dissociation from RFP-selector, and a low binding affinity. On the contrary, the expression of mTq2-tagged long and short pendrin isoforms was much lower, but cyan fluorescence on RFP-selector was evident with very low background fluorescence for Long-mCherry/Long-mTq2 (Fig. 4, panel g) and Short-mCherry/Short-mTq2 (Fig. 4, panel k). This observation suggests that, like the long isoform, the short pendrin isoform can also form a stable homodimer. Our result also suggests that heterodimer formation between the long and short pendrin isoforms is possible because mTq2 fluorescence was detectable on RFP-selector with relatively low background fluorescence for mCherry/Short-mTq2 and Short-mCherry/Long-mTq2 (Fig. 4, panels h and j compared to panels i and l). However, the heterodimer may be less stable compared to homodimers of the long or short isoforms judging from the low but noticeable background fluorescence (Fig. 4, panels h and j compared to panels g and k).

### HCO_3_^−^/Cl^−^ antiport assay for long and short pendrin isoforms

**Fig. 5:**
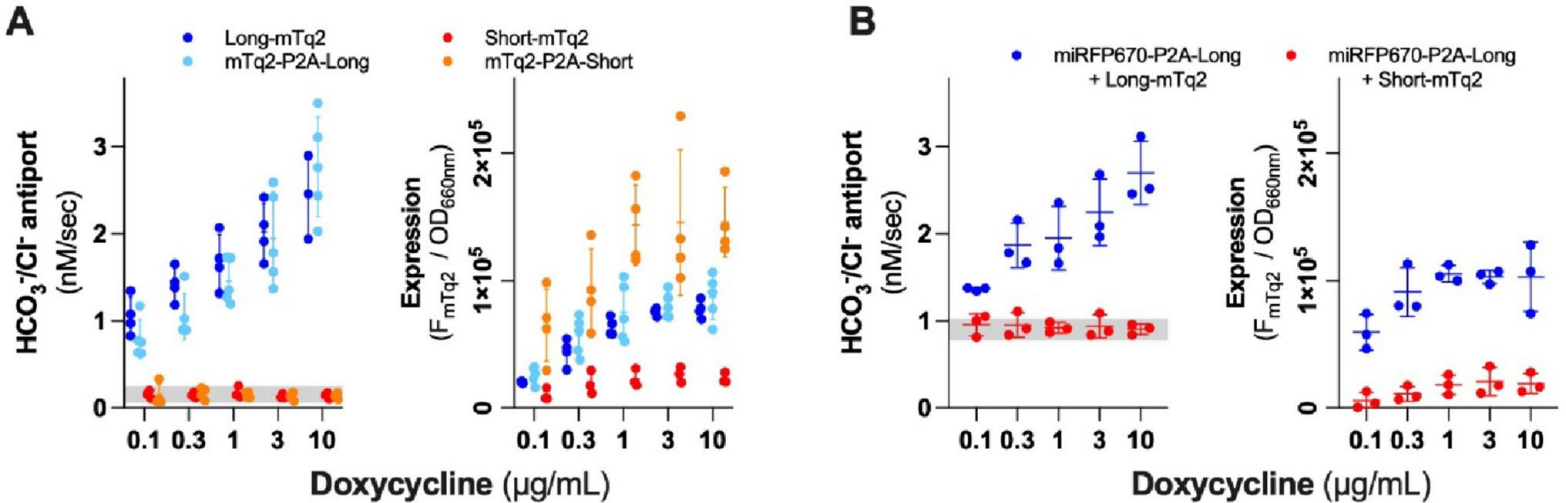
HCO_3_^−^/Cl^−^ antiport assay. HCO_3_^−^/Cl^−^ antiport rates were determined using a ratiometric pH-indicator SNARF-5 F (left panels). Total expression of the pendrin constructs was quantified by mTq2 fluorescence (F_mTq2_) divided by OD at 660 nm (right panels). Error bars indicate standard deviation. **A** The long or short human pendrin isoform with either a direct (C-terminal) or self-cleaved (P2A) mTq2 tag was singly expressed in HEK293T cells in a doxycycline (Dox)-dependent manner. A gray shade indicates mean ± standard deviation of non-induced negative control (0.15 ± 0.10 nM/sec, *n* = 15). **B** The long or short human pendrin isoform with a direct C-terminal mTq2 tag was expressed in a Dox-dependent manner in HEK293T cells that constitutively expressed miRFP670-P2A-linked long human pendrin isoform (miRFP670-P2A-Long). A gray shade indicates mean ± standard deviation of no-Dox control (0.90 ± 0.13 nM/sec, *n* = 6)

Since most transmembrane helices are lost, it is highly unlikely that the short pendrin isoform retains anion transport function. As confirmation, we performed an HCO_3_^−^/Cl^−^ antiport assay in HEK293T cells expressing the short or long human pendrin isoforms in a doxycycline (Dox)-dependent manner (Fig. 5A, left panel). The two pendrin isoforms were either directly or indirectly (via a P2A self-cleaving sequence) tagged with mTq2 to monitor protein expression (Fig. 5A, right panel). The long pendrin constructs (Long-mTq2 and mTq2-P2A-Long) showed similar Dox-dependent increases in transport activity (*p* > 0.05, F-test), affirming that C-terminal mTq2 tagging does not interfere with pendrin’s anion transport function. As anticipated, the anion transport activity of the short pendrin constructs (Short-mTq2 and mTq2-P2A-Short) was Dox-independent and indistinguishable from that of the no-Dox negative control (Fig. 5A, left panel, indicated by a gray shade). This negative observation may be partly ascribed to the reduced protein expression reflected in small F_mTq2_/OD_660nm_ values (Fig. 5A, right panel, red circles). Interestingly, the expression values of mTq2-P2A-Short (Fig. 5A, right panel, orange circles) were greater compared to those of the long pendrin constructs (Fig. 5A, right panel, blue and pale blue circles). This increased expression of mTq2-P2A-Short is intuitive, given the fact that smaller cDNA constructs tend to express better than larger ones in heterologous expression systems. The large discrepancy in expression between Short-mTq2 vs. mTq2-P2A-Short suggests that the short pendrin isoform is short-lived, possibly due to structural instability. We also confirmed the lack of transport activity and reduced protein expression for the mouse short pendrin isoform compared to the long isoform (Fig. S7A).

We next turned to the possibility that the short isoform may exert a regulatory effect on the long pendrin isoform upon heterodimerization and measured the HCO_3_^−^/Cl^−^ antiport activity in HEK293T cells constitutively expressing miRFP670-P2A-linked long human pendrin isoform (miRFP670-P2A-Long) and inducibly expressing either the long or short human pendrin isoform with a C-terminally attached mTq2 (Long-mTq2 or Short-mTq2) (Fig. 5B). The basal HCO_3_^−^/Cl^−^ antiport activity due to the constitutive expression of the long pendrin isoform (miRFP670-P2A-Long) without doxycycline is indicated by a gray shade. The HCO_3_^−^/Cl^−^ antiport activity further increased in a Dox-dependent inducible manner for Long-mTq2 as anticipated but not for Short-mTq2. We also measured the HCO_3_^−^/Cl^−^ antiport activity in HEK293T cells co-expressing miRFP670-P2A-Long constitutively and mTq2-P2A-linked short human pendrin isoform (mTq2-P2A-Short) in a Dox-inducible manner and confirmed the absence of any detectable regulatory effect of the short isoform on the long pendrin isoform (Fig. S7B).

### Genotype-phenotype correlation based on isoforms

**Fig. 6:**
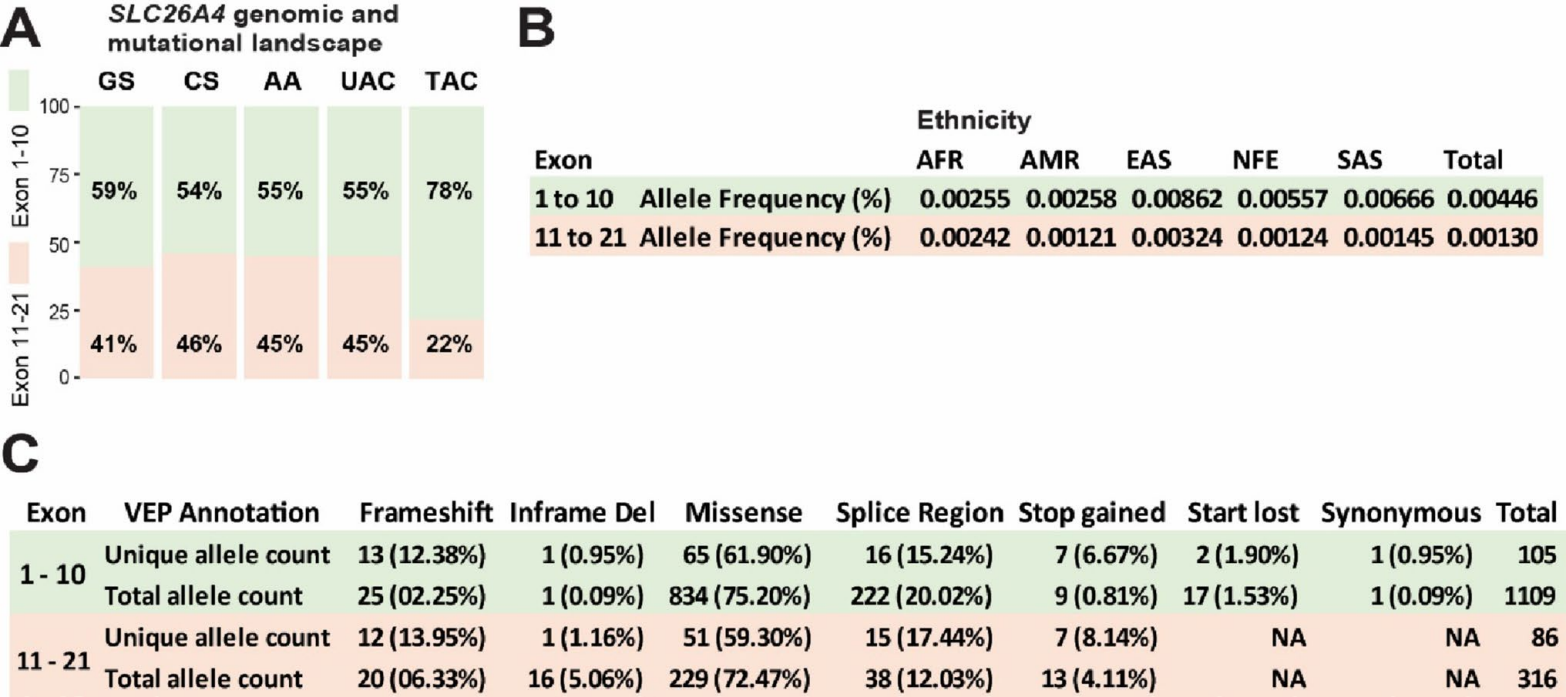
Genomic and mutational landscape of ***SLC26A4*** based on genome aggregation database (gnomAD) V2.1.1.** A** The genomic and coding sequence landscape of *SLC26A4*. **GS** (genomic sequence), exons + intron: total: 40,862 bp; exons 1–10: 24,078 bp (59%); exons 11–21: 16,748 bp (41%). **CS** (coding sequence): total: 2343 bp; exons 1–10: 1263 bp (54%); exons 11–21: 1080 bp (46%). **AA** (amino acid sequence): total: 780 aa; exons 1–10: 431 aa (55%); exons 11–21: 348 aa (45%). **UAC** (unique Pathogenic (P) and Likely Pathogenic (LP) allele count in gnomAD V2.1.1): total: 191; exons 1–10: 105 (55%); exons 11–21: 86 (45%). **TAC** (total P/LP allele count), sum of minor allele frequencies of P/LP variants: total: 1425; exons 1–10: 1109 (78%); exons 11–21: 316 (22%). **B** The prevalence of *SLC26A4* mutations based on ethnicity. P/LP *SLC26A4* variants from gnomAD V2.1.1. **AFR**: African/African American; **AMR**: Latino/Admixed American; **EAS**: East Asian; **NFE**: European (non-Finnish); **SAS**: South Asian. **C** Categorization by variant effect predictor (VEP) annotation based on exonic region

**Fig. 7:**
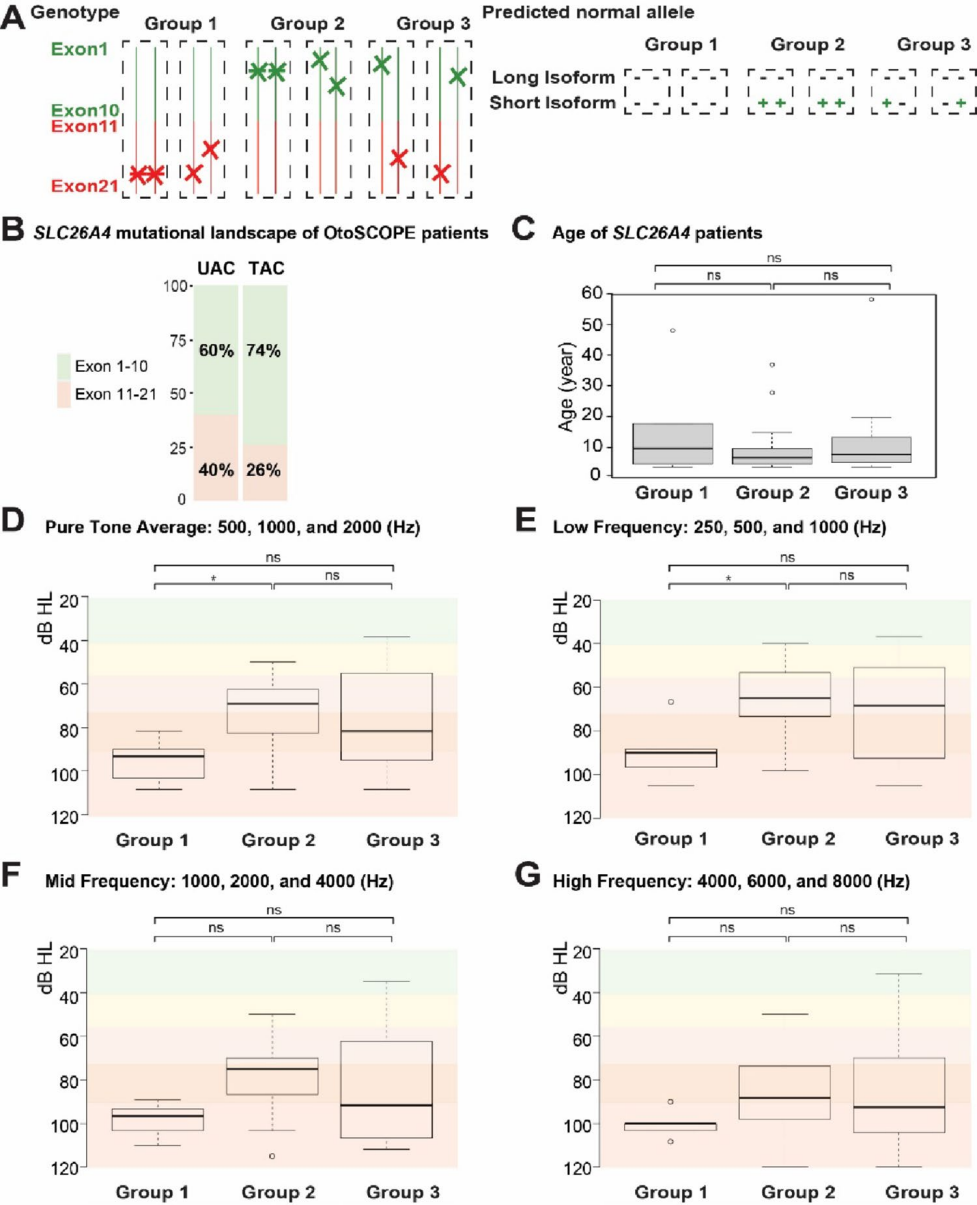
Genotype-hearing phenotype correlation in ***SLC26A4*** patients based on mutation location **A** Schematic of patient groups. Group 1 patients carry two pathogenic (P) and/or likely pathogenic (LP) variants in exons 11–21. Group 2 patients carry two P/LP variants in exons 1–10. In groups 1 and 2, patients are either homozygous or compound heterozygous. Group 3 patients carry one P/LP variant in exons 1–10 and a second P/LP variant in exons 11–21. All compound heterozygous variants are in trans. Expression of normal copies of the short isoform are predicted for groups 2 and 3. **B** *SLC26A4* mutational landscape of OtoSCOPE patients (*n* = 133) with two P/LP variants in trans in *SLC26A4*. UAC (total = 101) exons 1–10: 61 (60%); exons 11–21: 40 (40%); TAC: (total = 266): exons 1–10: 196 (74%); exons 11–21: 70 (26%). **C** Age of patients. The median age of patients in groups 1: 10.0 years; group 2: 10.2 years; group 3: 13.4 years. There is no significant difference in median age between groups. **D-G** Audio profile of patients. Group 1 (*n* = 5), Group 2 (*n* = 24) and Group 3 (*n* = 15) **D** Pure tone average (PTA): 500, 1000, and 2000 Hz. The medians for groups 1, 2 and 3 are 93.3 dB HL (profound), 69.2 dB HL (moderately severe), and 81.7 dB HL (severe), respectively. **E** Low frequency hearing: 250, 500, and 1000 Hz. The medians for groups 1, 2 and 3 are 90.0 dB HL (severe), 65.0 dB HL (moderately severe), and 68.3 dB HL (moderately severe), respectively. **F** Middle frequency hearing: 1000, 2000, and 4000 Hz. The medians for groups 1, 2 and 3 are 96.7 dB HL (profound), 75.0 dB HL (severe), and 91. 7 dB HL (profound), respectively. **G** High frequency hearing: 4000, 6000, and 8000 Hz. The medians for groups 1, 2 and 3 are 100.0 dB HL (profound), 88.3 dB HL (severe), and 92.5 dB HL (profound), respectively. All statistical analyses are done by one-way ANOVA. ns: not significant, *: *p* < 0.05

Although antiporter function of the short isoform was not demonstrable, if the short isoform plays a role in the biology of hearing independent of the canonical isoform, we hypothesized that patients carrying pathogenic variants in exons 11–21 would have a more severe hearing loss phenotype than patients with pathogenic variants in exons 1–10, since variants in the former group impact both pendrin isoforms while variants in the latter group affect only the long isoform (Fig. 7A). To evaluate this possibility, we re-classified the genomic and mutational landscape of *SLC26A4* at an isoform level. The genomic sequence (exons and introns) of *SLC26A4* spans 40,862 bp (exons 1–10, 24078 bp (59%); exons 11–21, 16748 bp (41%)) of which 2343 bp are coding sequence (exons 1–10, 1263 bp (54%); exons 11–21, 1080 bp (46%)). The long and short isoforms encode proteins of 780 and 349aa, respectively (Fig. 6A). We next investigated pathogenic (P) and likely pathogenic (LP) variants as reported in the genome aggregation database (gnomAD) and collated data by allele count (AC) and minor allele frequencies (MAF) across ethnicities (Figs. 6B-C and Table S1).

The total unique P/LP allele count (UAC) in gnomAD V2.1.1 is 191 variants, with 105 (55%) variants in exons 1–10 (and therefore impacting only the long isoform) and 86 (45%) variants in exons 11–21 (and therefore impacting both the short and long isoforms). These variants generate a total P/LP allele count (TAC) of 1425, with 1109 alleles (78%) associated with variation in exons 1–10 and 316 alleles (22%) associated with variation in exons 11–21 (Fig. 6A). Thus, although the UAC mirrors the aa lengths of exons 1–10 vs. exons 11–21, the TAC is ~ 4 times higher in exons 1–10 (Fig. 6A).

By ethnicity, in the admixed American (AMR), East Asian (EAS), non-Finnish European (NFE), and South Asian (SAS) populations, the TAC is similarly weighted to exons 1–10, while for the African (AFR) it is not (Fig. 6B and Table S1). These ethnic differences in TAC are reflected in the aggregate allele frequencies for P/LP alleles. For example, in the NFE population, P/LP variants are present with an aggregate frequency of 0.00862 across exons 1-10, implying that 1 of 116 persons of this ethnicity carries a P/LP variant in this portion of the long isoform of pendrin. However, only 1 in 313 NFE persons is a carrier of a P/LP variant across exons 11–21, which would impact both the short and long isoforms of the protein. Variable Effect Predictor (VEP) annotation shows that most of the difference in TAC for P/LP variants in exons 1–10 as compared to exons 11–21 is associated with missense mutations (Fig. 6C).

In our database of 6000 + patients with hearing loss who have undergone comprehensive genetic testing, we identified 133 patients with two P/LP variants in *SLC26A4* (74% in exons 1–10; 26% in exons 11–21) (Fig. 7B and Table S1). After excluding patients who were three years of age or younger, since *SLC26A4*-related hearing loss is known to progress before age three, audiograms from 36 patients were reviewed together with eight additional audiograms identified in publications (Mey et al. 2019). These 44 patients were classified in three groups by variant position: Group 1 - two P/LP variants in exons 11–21; Group 2 - two P/LP variants in exons 1–10; and Group 3 - one P/LP variant in exons 1–10 and one P/LP variant in exons 11–21. Analysis of binned audiograms based on variant location showed no significant difference between groups based on age (Fig. 7C). However, pure tone averages (PTAs) and low frequency thresholds were consistently better in patients in Group 2 as compared to patients in Group 1, suggesting that variants impacting both long and short isoforms induce a more severe phenotypic than mutations affecting the long isoform only (Figs. 7D-G). These results suggest that the short isoform plays a role in auditory function.

## Discussion

Determining the cell-type isoform diversity and their abundance under a physiological condition is essential for understanding the molecular mechanisms underlying various diseases. Isoform diversity is a common feature of neural and sensory organs as it offers an exquisite means of affording functional variability. By way of example, in the cochlea several studies illustrate the interrelationship between isoform-specific gene expression profiles and discrete aspects of auditory function. Two MYO15A isoforms, for example, are expressed in cochlear hair cells through alternative mRNA splicing: a short isoform (MYO15A-S, also known as MYO15A-2) localizes to the row 1 stereocilia tips, while a long isoform (MYO15A-L, also known as MYO15A-1) includes an additional 133 kDa N-terminal domain encoded by a specific large exon 2 and localizes to the row 2 stereocilia tips. The former is essential for stereocilia development and delivers an elongation-promoting complex (EC) to the tips of hair cells, while the latter regulates the size of shorter stereocilia postnatally, acting to tune their structure (Fang et al. 2015; Moreland et al. 2025). Clinically, pathogenic mutations in the unique N-terminal region of MYO15A-L often lead to an audioprofile characterized by better residual low frequency hearing whereas mutations affecting the shared motor or tail domains impact both isoforms and cause severe-to- profound congenital deafness. Another example is Otoferlin, a key protein for synaptic transmission in IHCs. Its long isoform drives synaptic vesicle fusion and exocytosis, while the shorter isoforms are involved in endocytic membrane recycling. The clinical consequence of deleterious mutations can range from severe-to-profound congenital deafness (DFNB9, OMIM #601071) to hidden hearing loss (Liu et al. 2023a, b).

While these two examples focus on sensory hair cells, given the complexity of the cochlea, it is reasonable to assume the extent of isoform diversity impacts non-sensory cells. To explore this possibility, we combined precise single-cell isolation using a micropipette-based technique with single-cell short-read (Illumina) and long-read (Nanopore) sequencing. We identified a novel short isoform of pendrin in two distinct subtypes of pendrin-expressing cell, SC and RC (Figs. 2 and 3C-D).

The long canonical isoform of pendrin is encoded by *SLC26A4* and spans 21 exons, which are translated into a 780-amino acid protein with 14 transmembrane domains; both the amino and carboxy termini are intracellular. The protein homodimerizes to form an antiporter for bicarbonate (HCO_3_^−^) and chloride (Cl^−^) ions that plays a crucial role in maintaining endolymph homeostasis in the inner ear (Liu et al. 2023a, b). The novel short isoform, that we confirmed to be expressed in both murine and human tissues, is transcribed using a conserved Kozak sequence in intron 10 and includes exons 11–21 of the canonical isoform (Fig. 2E, F and S4). It has three transmembrane domains and an intracellular STAS domain, suggesting that it may be needed for protein trafficking and protein-protein interactions (Fig. 3A-B). Using C-terminal antibodies, we confirmed the expression of the long and short isoform in both cochlea and kidney (Fig. 3E and S5A). Unexpectedly, in *Slc26a4*-/- mice the C-terminal antibodies detected the short isoform only in the cochlea (Fig. 3E) and not in the kidney (Fig. S5). It is noteworthy that the long and short isoforms of pendrin exhibit organ-specific differences in size, likely reflecting post-translational modifications (Ikegami et al. 2014). In the cochlea, we also observed cell-type-specific differences in isoform expression (Fig. 3D, F).

Despite the lack of the entire N-terminal cytosolic and most transmembrane regions, the short pendrin isoform can form homodimers and, presumably, heterodimers with the canonical long isoform (Fig. 4). To determine whether the short isoform has functional or regulatory role, we performed HCO_3_^−^/Cl^−^ antiport assays for the short pendrin isoform in the presence and absence of the canonical long isoform (Figs. 5 and S7). The absence of HCO_3_^−^/Cl^−^ antiport activity in the short isoform seems intuitive, but we also did not find any detectable modulatory effect of the short isoform on the anion transport function on the long pendrin isoform. However, since the expression level of the short pendrin isoform was low possibly due to structural instability, these negative observations should be taken with caution. It is possible that the short isoform may be stabilized *in vivo* by binding to its interacting partners.

Complex biological systems are composed of interacting proteins, which are crucial for cellular function. Several members of the Slc26 family form multi-protein complexes with the cytoskeleton, PDZ protein domain, and protein kinases (Xu et al. 2022; Lohi et al. 2003). AlphaFold 2.0-based predictions indicate that the long (Fig. S6A) and short (Fig. S6B) isoforms of SLC26A4 complex with carbonic anhydrase 13 (CA13) near the STAS domain, which we confirmed with colocalization experiments (Figs. S6C-E). Moreover, recent studies have shown that pendrin directly interacts with other interacting partners, such as AP2 µ2 (Lee et al. 2024) and IQGAP1 (Xu et al. 2022). IQGAP1 is a scaffolding protein involved in the regulation of signal transduction, cytoskeleton, cell adhesion and cell cycle (White et al. 2012; Hedman et al. 2015). Functional studies indicate that the interaction of IQGAP1 with the pendrin C-terminus enhances Cl^−^/HCO_3_^−^ antiporter activity. Therefore, it is possible that the short isoform could modulate Pendrin/IQGAP1 protein complex formation, thereby regulating activity. Alternatively, another interacting protein may be required for the short isoform to exert a modulatory effect on the long isoform. Further research is needed to test whether the short isoform indirectly impacts the function of the long isoform.

Our data suggest that the short isoform may play an important role in the auditory function. Reclassification of the genomic and mutational landscape of *SLC26A4* to investigate isoform-based phenotype-genotype associations shows that while the distribution of unique P and LP variants is almost equal when exons 1–10 are compared to exons 11–21 (55% vs. 45%), TAC is strikingly different (78% vs. 22%), suggesting more founder mutations in exons 1–10 (Figs. 6 and 7). Additionally, patients with two P and LP variants in exons 11–21 have higher PTA and low frequency thresholds compared to patients with variants in exons 1–10 (Fig. 7).

This study has two limitations. First, due to the difficulty in cell isolation, the number of cells we studied is small. Second, we were unable to define a specific role for the short pendrin isoform. Cochlear western blotting data show that *Slc26a4*^−/−^ mice express the short isoform but not the long isoform, indicating that expression of the short isoform does not support normal cochlear operation, and our *in vitro* data confirmed absence of transport function in the short isoform. Further studies will be required to clarify the role of the short pendrin isoform in the cochlea and the physiological significance of cell-specific isoform expression for normal inner ear function.

In summary, we have identified a novel short isoform of pendrin that does not have the antiporter function characteristic of the canonical long isoform. While its function remains to be determined, based on phenotype-genotype data, it may play a role in auditory function. As such, we believe that isoform-specific mouse models of *Slc26a4* should be generated to clearly define the role of both isoforms in the biology of hearing and deafness. This type of study could provide novel insights into the molecular mechanisms that drive the functional complexity of pendrin.

## Methods

### Animals

In this study we used 1 month old wildtype (WT) mice and *Slc26a4*^+/−^, *Slc26a4*^−/−^ on a 129S6 background (Everett et al. 2001). For scRNA-seq and RT-PCR experiments 1 month old WT mice were used (*n* = 55 and *n* = 5 respectively). For western blotting experiments, male *Slc26a4*^+/−^ (*n* = 3), *Slc26a4*^−/−^ (*n* = 3), and WT mice (*n* = 3) were used. For immunofluorescence experiments, *Slc26a4*^−/−^ (*n* = 3) and WT mice (*n* = 3) were used. All mice were maintained under temperature-controlled (22–23 °C), light-controlled (12-hour light cycle), and humidity-controlled (40%−60%) conditions with free access to food and water. All work was approved by the University of Iowa Institutional Animal Care and Use Committee (IACUC). Before cochlear dissection, animals were euthanized according to American Veterinary Medical Association guidelines (Leary S 2013).

### Cochlear lateral wall dissection, single-cell isolation and full-length cDNA preparation

The temporal bone was dissected, and the encapsulating bone was removed, the stria vascularis (SV) tissue was dissected from the cochlear membranous labyrinth. The isolated SV was incubated for 5 min at 37 °C with collagenase from clostridium histolyticum (C5138; Sigma Aldrich) solution at a working concentration of 0.3 mg/500µl. Cells were then dissociated by gently pipetting up and down and transferred to a glass microscope slide, which was placed under an inverted microscope equipped with two 3D micromanipulators, each driving a pulled-glass micropipette attached to a nitrogen gas-powered Pico-Injector to control aspiration pressure. The round-shaped cells were identified and aspirated, one at a time, into the first pulled-glass micro pipette in a slow and controlled manner. The aspirated cell was then transferred to a fresh glass microscope slide containing DPBS to wash free residual debris. Finally, the washed single-cell was aspirated using a second clean pulled-glass micropipette and ejected into a 0.2-ml thin-walled PCR tube containing lysis buffer, 0.2% Triton X-100, oligo-dT primer, dNTP mix, and RNase inhibitor (10777019; Invitrogen) (Picelli et al. 2014).

Reverse transcription (RT) and PCR amplification were performed successively to create complementary DNA (cDNA) libraries using the SMART-Seq2 protocol and Superscript III Reverse Transcriptase (18080093; Invitrogen). After cDNA purification using Ampure XP beads (17022200; Beckman Coulter), cDNA quality was checked using the Agilent 2100 Bioanalyzer and high-sensitivity DNA chip kits (5067 − 4626; Agilent Technologies, Santa Clara, CA). All full-length cDNAs showed a peak at ~ 1.5–2 kb.

### Library preparation and single-cell RNA-sequencing (scRNA-seq)

#### Illumina short-read sequencing library preparation

Nextera XT DNA sample preparation kits (15032354; Illumina), including Tn5 enzymes for tagmentation, were used with Nextera XT index kits (15055294; Illumina) for multiplexing using 0.125 ng of DNA input. The adapter-ligated fragments underwent amplification (14 cycles) and purification using AMPure XP beads. After purification, single-cell cDNA libraries were run on the Agilent 2100 Bioanalyzer high-sensitivity DNA chips to check tagmentation. All libraries showed a peak at ~ 300–800 bp after tagmentation. Based on index balance, single-cell cDNA libraries were combined in equimolar concentrations. The concentration of pools was measured with the Qubit dsDNA HS Assay Kit (Q32854; Invitrogen) on a Qubit 2.0 Fluorometer (Q32866; Invitrogen). Before sequencing, samples were cleaned twice with AMPure XP bead to remove free primers.

#### Illumina short-read sequencing

Pooled single-cell cDNA libraries were sequenced on a single lane of an Illumina HiSeq 4000 system with 150-bp paired-end chemistry (Illumina) at the Genomics Division of the Iowa Institute of Human Genetics (IIHG).

#### Oxford Nanopore MinION long-read sequencing library preparation

In addition to the short-read Illumina sequencing, we also sequenced full-length cDNA from the same cells using the MinION long-read sequencer (Oxford Nanopore Technologies; ONT). Unlike Illumina short-read sequencing, which requires fragment sizes (150–800 bp), Oxford Nanopore sequencing generates single reads that span the length of an mRNA transcript. For 1D MinION long-read sequencing, the full-length cDNA product of each cell had unique index primers for multiplexing and was amplified with KAPA HiFi Readymix 2 × (7958935001; Kapa Biosystems) using the ISPCR primers and 20 amplification cycles, as previously described (Byrne et al. 2017). The input cDNA was 3 µg from each pool (750 ng/sample for four samples).

NEBNext Ultra II End Prep Enzyme Mix (E7646A; NEB) and NEBNext End Prep Reaction Buffer (E7647A; NEB) were used to assemble each pool. After mixing cDNA and NEB End Prep by flicking, the mixture was placed in a thermocycler at 20 °C for 30 min and then at 65 °C for 20 min. To prepare the adapter-ligation reaction, End-prepped DNA, Adapter mix 1D of Ligation Sequencing Kit 1D (AMX 1D, SQK-LSK108; ONT) and Blunt/TA Ligase Master Mix (M0367S; NEB) were mixed and incubated at room temperature for 20 min. After purifying the reaction using AMPure XP beads, an Adapter Bead Binding Buffer (ABB, SQK-LSK108; ONT) was used to rinse the reaction. Elution Buffer (ELB, SQK-LSK108; ONT) was used to elute DNA. The DNA, priming buffer (RBF, SQK-LSK108; ONT), and Library Loading Bead (LLB, EXP-LLB001; ONT) were mixed to prepare the library.

#### Oxford Nanopore long-read sequencing

The four barcoded single-cell libraries were combined into a single batch. We ran 10 batches of pooled libraries. A total of 40 cells from the lateral wall tissue were used, including intermediate cells (IMC), spindle cells (SC), root cells (RC), and marginal cells (MC), as previously described (Koh et al. 2023) following Illumina sequencing and analysis. Among them, 20 cells expressing *Slc26a4* were used for this study. The single R9.4 flow cells were sequenced on the nanopore MinION sequencer for 48 h at the Molecular Otolaryngology and Renal Research Laboratories (MORL). MinKNOW software (ONT) was used to monitor the sequencing and library quality.

### Extraction of cochlear total RNA, RT-PCR

The cochlear lateral wall, including SV tissue, was dissected, and stored at −80 °C for later RNA extraction. To extract RNA, 150 µl of buffer RLT (1.5 µl of β-mercaptoethanol) was added to the tissue, which was then homogenized with a Polytron homogenizer (Kinematica AG, Cincinnati, OH, USA, probe PT-DA 1205/2EC) to maximize yield and quality of total RNA. RNA was purified using RNeasy Plus Universal Mini Kit (73404; Qiagen) following the manufacturer’s instructions, adding a proteinase K and DNase treatment steps Total RNA was eluted in 20 µl of elution buffer. Reverse transcription (RT) was performed using the SuperScript III First-Strand Synthesis System (18080051; Invitrogen) following the manufacturer’s instructions. RNase H was added to the mix to remove RNA and incubated at 37 °C for 20 min.

The short- and long-isoforms cDNA were detected by PCR using EmeraldAmp GT PCR Master Mix (RR310A; Takara Bio). The forward primer for all mouse short isoforms was 5´-TCTGAGCTTCCTTTACCCCA- 3´ and the reverse primers were 5´-TCACAATCACAGCTGAGATGAG- 3´ (S1), 5´- CACAGTCAGTAGTGCAAATAAAAGG- 3´ (S2), and 5´ TCAGGATCTTCACCCCTTCAG- 3´ (S3). The forward primer for the long isoform was 5´- CATTGCTGTGGTGGCTTACG- 3´, and the reverse primers were 5´- CACAGTCAGTAGTGCAAATAAAAGG- 3´ (L1) and 5´- ATCTTCACCCCTTCAGGCTC- 3´ (L2) (see Fig. 2D). Human total cDNA from adult normal thyroid (C1234265; BioChain) and kidney (C1234142; BioChain) were used to assess the expression of both isoforms using the following primers: Short isoform: forward primer was 5´- TCTGAAGCTTCCTTTTACCCC- 3´, and the reverse primers were 5´- TCACAATCGCAGCAGAGATGAT- 3´ (S1), 5´- CACAGTCAACAGTCCAAATATAAGG- 3´ (S2), and 5´ TAAGAATCTTCACTCCTTGAG- 3´ (S3). Long isoform: forward primer was 5´- CATCGCTGTGGTGGCTTATG- 3´, and the reverse primers were 5´- CACAGTCAACAGTCCAAATATAAGG- 3´ (L1) and 5´- ATCTTCACTCCTTGAGGTTC- 3´ (L2) (see Fig. S4). The PCR reaction was denatured at 95 °C for 1 min, followed by 40 cycles of 95 °C for 15 s, 59 °C for 15 s and 72 °C for 30 s, with a final extension step at 72 °C for 7 min.

### Generation of pendrin peptide-specific antibodies

We generated anti-pendrin antisera by immunizing rabbits with synthetic peptides encompassing the mouse pendrin’s C-terminal amino acid sequences and N-terminal amino acid sequences (NCBI NP_035997.1). Anti-pendrin antisera were generated by immunizing rabbits with a synthetic peptide EELDVQDEAMRRLAS corresponding to the C-terminal region (amino acid 766–780) of mouse pendrin (NCBI NP_035997.1; the same site used in other studies (Porra et al. 2002; Choi et al. 2011)). For the N-terminal region, anti-pendrin antisera were generated by immunizing guinea pigs with a synthetic peptide QQRERRLPERRTLR corresponding to the N-terminal area (amino acid 33–47). Final antisera were purified by peptide antigen affinity column; antibodies that were bound specifically to the peptide antigen were retained upon loading of the sera while impurities and non-specific IgG were discarded in the flow-through. Antibodies were purified without regard to antibody class, isotype or size selection.

### Tissue lysate preparation for western blotting

To prepare kidney tissue for western blotting, animals were perfused with 1X PBS and then the kidney was harvested, and its capsule carefully removed. Next, the kidney was cut into small pieces and placed in a Protein LoBind Tube (022431081; Eppendorf). Tissue samples were frozen at −80 °C, weighed rapidly (50–1200 mg tissue/1 ml) and homogenized using a Cole-Parmer PTFE tissue grinder (UX-44468-06; Cole-Parmer) in ice-cold homogenization buffer (10mmol/L triethanolamine [TEA] (90279; Sigma) and 250mmol/L sucrose (S24060-500; RPI), adjusted to pH7.6 with HCl) containing protease inhibitor cocktail set I (539131; Calbiochem) and phenylmethylsulfonyl fluoride (PMSF) (P7626; Sigma) as protease inhibitors. For the mouse cochlea, 1 M NaCl, 20mM Tris, 5mM EDTA buffer containing 1mM PMSF, 1% SDS, protease inhibitor cocktail (A32953; Pierce) was used to extract the protein. Homogenates were centrifuged at 4,000 x g at 4 °C for 10 min, and supernatants were carefully placed into an ice-cold 1.5 mL tube. The total protein concentrations were measured by a Pierce bicinchoninic acid assay (BCA) protein assay kit (23227; Thermo Scientific).

### Western blotting

Cochlear homogenates were prepared in Laemmli sample buffer with BME as described previously (Affortit et al. 2021). 20 µg of total protein homogenate were fractioned by electrophoresis in 10% Mini-PROTEAN TGX gels and transferred onto nitrocellulose membrane. Blots were incubated with antibodies recognizing anti- C-Term Pendrin (1:100), β-actin (1:10000, Sigma-Aldrich #A1978 RRID: AB-476692) served as a loading control. The secondary antibodies used were horseradish peroxidase-conjugated goat anti-mouse IgG (1:3000, Jackson ImmunoResearch #115-001-003 RRID: AB-2338443), or goat anti-rabbit IgG (1:3000, Jackson ImmunoResearch #111-001-003 RRID: AB-2337910). Image scans of Western blots were used for semi-quantitative analysis. Western blot analysis required 12 additional animals (24 cochleae) per age and strain. Each experiment with a pool of 8 cochleae (4 animals per sample) was performed in biological and technical triplicate.

Western blotting for kidney tissue was performed as described previously (Nizar et al. 2018). Total membrane proteins of each lane were visualized and quantified by Coomassie Blue Staining using GelCode Blue Safe Protein Stain (2459; Thermo Scientific). Following the gel electrophoresis, proteins were transferred to 0.2 µM polyvinylidene difluoride (PVDF) membranes (1620174; Bio-Rad Laboratories) for western blotting. After blocking with 5% nonfat milk powder in phosphate-buffered saline/0.1% tween 20 (PBST) for 1 h at room temperature, the membrane was incubated with the primary antibody (rabbit anti-pendrin) at a 1:10,000 concentration overnight at 4 °C. Then, the membrane was washed in PBST (3 × 5 min) and incubated with the secondary antibody, goat anti-rabbit IgG-HRP (4030-05; SouthernBiotech), at a 1:20,000 concentration for 1 h at room temperature. The blots were detected using SuperSignal West Pico PLUS Chemiluminescent Substrates (34580; Thermo Scientific) and visualized using a Bio-Rad Gel Doc EQ System w/Universal Hood II Imaging System (Bio-Rad Laboratories).

### Tissue section preparation for immunofluorescence staining

In preparation for immunofluorescence staining, cochlea and kidney were fixed in 4% paraformaldehyde (15710-S; Electron Microscopy Sciences). Cochlea tissues were decalcified for 48 h in EDTA solution (0.5 M, pH 7.4) and then cryopreserved by progressive incubation from a solution of 20% sucrose to pure Tissue-Tek optimum cutting temperature (OCT, 4583; Sakura Finetek). Tissues were embedded in fresh OCT and stored at −80 °C before sectioning using a Leica Microtome RM2135 (Leica, Germany). Kidney tissues were cryopreserved in 10% sucrose, gradually increasing concentrations to 20%, and 30%. Tissues were then put in the OCT, immediately frozen in liquid nitrogen, and sectioned using Leica Microtome RM2135.

### Immunofluorescence staining

Frozen cochlear sections were blocked and permeabilized with a 30% normal donkey serum solution and 0.3% Triton X-100 in 1X PBS. Immunocytochemistry was performed in cochlear transversal cryostat sections from WT and *Slc26a4*^−/−^ mice aged one month. Frozen kidney sections were blocked and permeabilized with 5% normal goat serum and 0.2% Triton X-100 for 30 min before overnight incubation with anti-pendrin primary rabbit antibody (1:100, #2842), anti-pendrin primary guinea pig antibody (1:100), anti-carbonic anhydrase 13 primary rabbit antibody (1:100, Proteintech Cat# 16696-1-AP, RRID: AB_1850972). Secondary antibody Alexa Fluor 488 - goat anti-rabbit (A-11008; RRID AB_143165) or Alexa Fluor 647 goat anti-guinea pig (A-21450; RRID AB_2735091) were incubated for 2 h at room temperature. In the cochlear sections, as a control, filamentous actin was labeled with phalloidin conjugated to Alexa 568 at a 1:1000 concentration for 2 h. Mounting was performed using ProLong Diamond mounting medium with DAPI (Life Technologies). Images of the cochlea and kidney sections were collected at 10x-63x on a Zeiss LSM 710 confocal microscope (Zeiss, Germany). ZEISS ZEN 3.3 (blue edition); ImageJ Fiji (Schneider, Rasband, and Eliceiri 2012) software was used.

### Generation of stable cell lines

Stable cells that express recombinant human and mouse pendrin constructs (both long and short isoforms) in a doxycycline dosage-dependent manner were established in HEK293T cells as previously described (Kuwabara et al. 2018; Wasano et al. 2020; Kojima et al. 2021; Takahashi et al. 2024; Takahashi and Homma 2024). Briefly, cDNAs encoding human and mouse pendrin (long and short isoforms) fused with a mTurquoise2 (mTq2) tag on the C-terminus were cloned into a pSBtet-Pur vector (60507, Addgene) using SfiI sites. Also, cDNA of human pendrin (long and short isoforms) with N-terminal mTq2 and P2A sequence were cloned into pSBtet-Pur as above. For co-expression of human pendrin long isoform in antiport assays, cDNA of human pendrin long isoform with N-terminal miRFP670 and P2A sequence was cloned into a pSBtet-Bla vector (60510, Addgene) using SfiI sites. For co-precipitation assay, human pendrin (both long and short isoforms) with C-terminal mCherry tags were cloned into a pSBtet-Bla vector. Plasmid encoding mTq2 alone was previously generated (Wasano et al. 2020). These constructs were introduced to HEK293T cells together with pCMV(CAT)T7-SB100 (34879, Addgene) using Effectene transfection reagent (301425, Qiagen). Transfected cells were selected in a DMEM medium (11965, Thermo Fisher Scientific) supplemented with 10% FBS and 1 µg/mL puromycin (A11138, Thermo Fisher Scientific) and/or 10 µg/mL blasticidin (A11139, Thermo Fisher Scientific) accordingly. The complete cDNA sequences of the human and mouse pendrin constructs used for the functional study are provided as supplementary information (Fig. S8).

### Co-precipitation assay

A co-precipitation assay was performed as described in detail in a previous study (Kojima et al. 2021). Briefly, cells co-expressing mCherry-tagged long or short human pendrin isoform and mTq2-tagged long or short human pendrin isoforms (or mTq2 as negative control) were lysed in a buffer containing 150 mM NaCl, 20 mM n-dodecyl-β-D-maltoside (DDM), 1 mM EDTA, 1 mM dithiothreitol (DTT), 20 mM HEPES and 50 µg/ml leupeptin (pH 7.5). After centrifugation (13,000 g for 10 min at 4 °C), the supernatants were collected and incubated with RFP-selector (N0410-L, NanoTag Biotechnologies) for 30 min at 4 °C on a nutator. The unbound fractions were further incubated with GFP-selector (N0310, NanoTag Biotechnologies) for 30 min at 4 °C on a nutator. The fluorescence of the RFP- and GFP-selector beads were imaged using an inverted epifluorescence microscope (DM IRB, Leica), and the obtained images were analyzed using ImageJ Fiji (Schneider, Rasband, and Eliceiri 2012).

### HCO_3_^−^/Cl^−^ antiport assay

The procedure of HCO_3_^–^/Cl^–^antiport assay was described in detail in a previous study (Wasano et al. 2020). Briefly, cells were loaded with a ratiometric pH indicator, SNARF-5F (S23923, Thermo Fisher Scientific), in a high Cl^***−***^ buffer (140 mM NaCl, 4.5 mM KCl, 1 mM MgCl_2_, 2.5 mM CaCl_2_, 20 mM HEPES, pH 7.4, 320 mOsmol/L) for 30 min in the presence of 5% CO_2_ at room temperature. After being transferred to wells in a 96-well plate (~ 1.5 × 10^5^/well), HCO_3_^−^/Cl^−^ antiport was initiated by an automated injection of a low Cl^−^ buffer (125 mM Na-gluconate, 5 mM K-gluconate, 1 mM MgCl_2_, 1 mM CaCl_2_, 20 mM HEPES, 25 mM NaHCO_3_, 5% CO_2_) in a plate reader equipped with dual top PMTs (Synergy Neo2, Agilent/BioTek). The fluorescence of SNARF-5F was measured in a time-dependent manner using the optical configuration described in the previous study (Wasano et al. 2020).

### Bioinformatic analyses

#### Transcript structure analyses

##### Illumina short-read sequencing

To identify alternative splicing events, alternative transcription start sites (TSSs) and transcription end sites (TESs), read alignment and transcript assembly were done using a customized Bash script in three steps. First, Illumina raw sequences were aligned to the mm10 genome (GRCm38) using the STAR (Spliced Transcripts Alignment to a Reference) (Dobin et al. 2013) aligner. Second, SAMtools (Sequence Alignment/Map) (Danecek et al. 2021; Li et al. 2009) was used to create and sort BAM files. Third, the mapped reads were assembled into the transcripts using Cufflinks (Trapnell et al. 2012). To visualize splicing junctions, Sashimi plots (Katz et al. 2015) in Integrative Genomics Viewer (IGV) (Robinson et al. 2011; Thorvaldsdottir et al. 2013) were used with sorted BAM files from Illumina sequencing.

##### Nanopore long-read sequencing

We used the Mandalorion pipeline (Byrne et al. 2017) to align the sequencing reads from FASTQ files to the mm10 genome (GRCm38) and to quantify isoform diversity. TSSs, TESs, and alternative splicing events, including alternative splice sites, intron retention, and exon skipping, were visualized IGV. A customized R script was used to enable a quantitative comparison of isoform usage across samples, incorporating the output of the Mandalorion pipeline.

##### Kozak consensus sequence of isoforms

We used ggseqlogo in R (Wagih 2017) to draw sequence logos.

#### Cell type filtering

Of the 20 cells expressing *Slc26a4* in Illumina sequencing, only 12 cells expressed *Slc26a4* in nanopore sequencing. To gain insights into the cell-type specific S*lc26a4* isoform expression we filtered 12 high quality cell based on gene expression levels using cell type defining gene *Anxa1* for the spindle cells and *Epyc* for the root cells (Koh et al. 2023; Gu et al. 2020). Based on this filtering, six cells were classified as spindle cells (mean raw count 8580 and 0.7 for *Anxa1* and *Epyc* gene respectively) and 5 were classified as root cells (mean raw count 3.6 and 2269 for *Anxa1* and *Epyc* gene respectively) and one cell was excluded due to the absence of strong markers expression. These filtered cells were used to measure S*lc26a4* long and short isoform expression.

##### Genome aggregation database (gnomAD) and deafness variation database (DVD) data

Pathogenic (P) and likely pathogenic (LP) *SLC26A4* variants (*n* = 101) were collected from gnomAD (Karczewski et al. 2020) 2.1.1. In addition, P/LP *SLC26A4* variants (*n* = 90) were also obtained from our in-house Deafness Variation Database (DVD) v9 (https://deafnessvariationdatabase.org/).

##### Audiometric data

The dataset used in this study consisted of 50 original audiograms collected from 36 MORL patients, as well as eight audiograms from publications. The typical audiogram included data for seven frequencies: 250 Hz, 500 Hz, 1 kHz, 2 kHz, 4 kHz, 6 kHz, and 8 kHz (Taylor et al. 2013; Thorpe et al. 2021). In all cases, the better hearing ear was used at each frequency to generate a composite audiogram, with missing thresholds between 250 Hz and 8 kHz imputed via cubic spline interpolation (**≥** 4 frequencies available) or linear interpolation (**≤** 3 frequencies available). Pure tone average (PTA) was calculated as the average of 500 Hz, 1000 Hz, and 2000 Hz thresholds. In addition, we determined low, middle and high frequency averages, which were defined as 250–500-1000 Hz, 1000-2000-4000 Hz, and 4000-6000-8000 Hz, respectively. The severity of hearing loss was defined by thresholds as profound (> 90 dB HL), severe (71–90. dB HL), moderately severe (56–70 dB HL), moderate (41–55 dB HL), and mild (20–40 dB HL) (Smith et al. 2005). 

## Supplementary Information

Below is the link to the electronic supplementary material.

Supplementary Material 1

Supplementary Material 2

Supplementary Material 3

Supplementary Material 4

Supplementary Material 5

Supplementary Material 6

Supplementary Material 7

Supplementary Material 8

Supplementary Material 9

Supplementary Material 10

## Data Availability

The data supporting this study’s findings are currently being deposited in NCBI’s Gene Expression Omnibus to be accessible through a GEO Series accession number GSE299266 before publication. To review GEO accession GSE299266 go to https:/www.ncbi.nlm.nih.gov/geo/query/acc.cgi? acc=GSE299266and enter token kvchsqwgfxolvyv into the box.
