# Supplementary material for: Identification of a novel isoform of Slc26a4 by single-cell RNA-sequencing of pendrin-expressing cells in the cochlea
Source: Hum Genet. 2026 Jul 16;145(1):62. doi: 10.1007/s00439-026-02858-x (PMC13375699; doi:10.1007/s00439-026-02858-x)
Supplement: Supplementary file 1 — Supplementary Material 1 [file 439_2026_2858_MOESM1_ESM.docx]

**Supplementary Fig. 8. The cDNA sequences of the human and mouse pendrin constructs used in this study**

**Human Long-mCherry (hSLC26A4_long_isoform-linker-mCherry, used in Fig.4)**

**ATGGCAGCGCCAGGCGGCAGGTCGGAGCCGCCGCAGCTCCCCGAGTACAGCTGCAGCTACATGGTGTCGCGGCCGGTCTACAGCGAGCTCGCTTTCCAGCAACAGCACGAGCGGCGCCTGCAGGAGCGCAAGACGCTGCGGGAGAGCCTGGCCAAGTGCTGCAGTTGTTCAAGAAAGAGAGCCTTTGGTGTGCTAAAGACTCTTGTGCCCATCTTGGAGTGGCTCCCCAAATACCGAGTCAAGGAATGGCTGCTTAGTGACGTCATTTCGGGAGTTAGTACTGGGCTAGTGGCCACGCTGCAAGGGATGGCATATGCCCTACTAGCTGCAGTTCCTGTCGGATATGGTCTCTACTCTGCTTTTTTCCCTATCCTGACATACTTTATCTTTGGAACATCAAGACATATCTCAGTTGGACCTTTTCCAGTGGTGAGTTTAATGGTGGGATCTGTTGTTCTGAGCATGGCCCCCGACGAACACTTTCTCGTATCCAGCAGCAATGGAACTGTATTAAATACTACTATGATAGACACTGCAGCTAGAGATACAGCTAGAGTCCTGATTGCCAGTGCCCTGACTCTGCTGGTTGGAATTATACAGTTGATATTTGGTGGCTTGCAGATTGGATTCATAGTGAGGTACTTGGCAGATCCTTTGGTTGGTGGCTTCACAACAGCTGCTGCCTTCCAAGTGCTGGTCTCACAGCTAAAGATTGTCCTCAATGTTTCAACCAAAAACTACAATGGAGTTCTCTCTATTATCTATACGCTGGTTGAGATTTTTCAAAATATTGGTGATACCAATCTTGCTGATTTCACTGCTGGATTGCTCACCATTGTCGTCTGTATGGCAGTTAAGGAATTAAATGATCGGTTTAGACACAAAATCCCAGTCCCTATTCCTATAGAAGTAATTGTGACGATAATTGCTACTGCCATTTCATATGGAGCCAACCTGGAAAAAAATTACAATGCTGGCATTGTTAAATCCATCCCAAGGGGGTTTTTGCCTCCTGAACTTCCACCTGTGAGCTTGTTCTCGGAGATGCTGGCTGCATCATTTTCCATCGCTGTGGTGGCTTATGCTATTGCAGTGTCAGTAGGAAAAGTATATGCCACCAAGTATGATTACACCATCGATGGGAACCAGGAATTCATTGCCTTTGGGATCAGCAACATCTTCTCAGGATTCTTCTCTTGTTTTGTGGCCACCACTGCTCTTTCCCGCACGGCCGTCCAGGAGAGCACTGGAGGAAAGACACAGGTTGCTGGCATCATCTCTGCTGCGATTGTGATGATCGCCATTCTTGCCCTGGGGAAGCTTCTGGAACCCTTGCAGAAGTCGGTCTTGGCAGCTGTTGTAATTGCCAACCTGAAAGGGATGTTTATGCAGCTGTGTGACATTCCTCGTCTGTGGAGACAGAATAAGATTGATGCTGTTATCTGGGTGTTTACGTGTATAGTGTCCATCATTCTGGGGCTGGATCTCGGTTTACTAGCTGGCCTTATATTTGGACTGTTGACTGTGGTCCTGAGAGTTCAGTTTCCTTCTTGGAATGGCCTTGGAAGCATCCCTAGCACAGATATCTACAAAAGTACCAAGAATTACAAAAACATTGAAGAACCTCAAGGAGTGAAGATTCTTAGATTTTCCAGTCCTATTTTCTATGGCAATGTCGATGGTTTTAAAAAATGTATCAAGTCCACAGTTGGATTTGATGCCATTAGAGTATATAATAAGAGGCTGAAAGCGCTGAGGAAAATACAGAAACTAATAAAAAGTGGACAATTAAGAGCAACAAAGAATGGCATCATAAGTGATGCTGTTTCAACAAATAATGCTTTTGAGCCTGATGAGGATATTGAAGATCTGGAGGAACTTGATATCCCAACCAAGGAAATAGAGATTCAAGTGGATTGGAACTCTGAGCTTCCAGTCAAAGTGAACGTTCCCAAAGTGCCAATCCATAGCCTTGTGCTTGACTGTGGAGCTATATCTTTCCTGGACGTTGTTGGAGTGAGATCACTGCGGGTGATTGTCAAAGAATTCCAAAGAATTGATGTGAATGTGTATTTTGCATCACTTCAAGATTATGTGATAGAAAAGCTGGAGCAATGCGGGTTCTTTGACGACAACATTAGAAAGGACACATTCTTTTTGACGGTCCATGATGCTATACTCTATCTACAGAACCAAGTGAAATCTCAAGAGGGTCAAGGTTCCATTTTAGAAACGATCACTCTCATTCAGGATTGTAAAGATACCCTTGAATTAATAGAAACAGAGCTGACGGAAGAAGAACTTGATGTCCAGGATGAGGCTATGCGTACACTTGCATCCCGGGACCCACCGGTCGCCACCATGGTGAGCAAGGGCGAGGAGGATAACATGGCCATCATCAAGGAGTTCATGCGCTTCAAGGTGCACATGGAGGGCTCCGTGAACGGCCACGAGTTCGAGATCGAGGGCGAGGGCGAGGGCCGCCCCTACGAGGGCACCCAGACCGCCAAGCTGAAGGTGACCAAGGGTGGCCCCCTGCCCTTCGCCTGGGACATCCTGTCCCCTCAGTTCATGTACGGCTCCAAGGCCTACGTGAAGCACCCCGCCGACATCCCCGACTACTTGAAGCTGTCCTTCCCCGAGGGCTTCAAGTGGGAGCGCGTGATGAACTTCGAGGACGGCGGCGTGGTGACCGTGACCCAGGACTCCTCCCTGCAGGACGGCGAGTTCATCTACAAGGTGAAGCTGCGCGGCACCAACTTCCCCTCCGACGGCCCCGTAATGCAGAAGAAGACCATGGGCTGGGAGGCCTCCTCCGAGCGGATGTACCCCGAGGACGGCGCCCTGAAGGGCGAGATCAAGCAGAGGCTGAAGCTGAAGGACGGCGGCCACTACGACGCTGAGGTCAAGACCACCTACAAGGCCAAGAAGCCCGTGCAGCTGCCCGGCGCCTACAACGTCAACATCAAGTTGGACATCACCTCCCACAACGAGGACTACACCATCGTGGAACAGTACGAACGCGCCGAGGGCCGCCACTCCACCGGCGGCATGGACGAGCTGTACAAG**TAA

**Human Short-mCherry (hSLC26A4_short_isoform-linker-mCherry, used in Fig.4)**

**ATGATCGCCATTCTTGCCCTGGGGAAGCTTCTGGAACCCTTGCAGAAGTCGGTCTTGGCAGCTGTTGTAATTGCCAACCTGAAAGGGATGTTTATGCAGCTGTGTGACATTCCTCGTCTGTGGAGACAGAATAAGATTGATGCTGTTATCTGGGTGTTTACGTGTATAGTGTCCATCATTCTGGGGCTGGATCTCGGTTTACTAGCTGGCCTTATATTTGGACTGTTGACTGTGGTCCTGAGAGTTCAGTTTCCTTCTTGGAATGGCCTTGGAAGCATCCCTAGCACAGATATCTACAAAAGTACCAAGAATTACAAAAACATTGAAGAACCTCAAGGAGTGAAGATTCTTAGATTTTCCAGTCCTATTTTCTATGGCAATGTCGATGGTTTTAAAAAATGTATCAAGTCCACAGTTGGATTTGATGCCATTAGAGTATATAATAAGAGGCTGAAAGCGCTGAGGAAAATACAGAAACTAATAAAAAGTGGACAATTAAGAGCAACAAAGAATGGCATCATAAGTGATGCTGTTTCAACAAATAATGCTTTTGAGCCTGATGAGGATATTGAAGATCTGGAGGAACTTGATATCCCAACCAAGGAAATAGAGATTCAAGTGGATTGGAACTCTGAGCTTCCAGTCAAAGTGAACGTTCCCAAAGTGCCAATCCATAGCCTTGTGCTTGACTGTGGAGCTATATCTTTCCTGGACGTTGTTGGAGTGAGATCACTGCGGGTGATTGTCAAAGAATTCCAAAGAATTGATGTGAATGTGTATTTTGCATCACTTCAAGATTATGTGATAGAAAAGCTGGAGCAATGCGGGTTCTTTGACGACAACATTAGAAAGGACACATTCTTTTTGACGGTCCATGATGCTATACTCTATCTACAGAACCAAGTGAAATCTCAAGAGGGTCAAGGTTCCATTTTAGAAACGATCACTCTCATTCAGGATTGTAAAGATACCCTTGAATTAATAGAAACAGAGCTGACGGAAGAAGAACTTGATGTCCAGGATGAGGCTATGCGTACACTTGCATCCCGGGACCCACCGGTCGCCACCATGGTGAGCAAGGGCGAGGAGGATAACATGGCCATCATCAAGGAGTTCATGCGCTTCAAGGTGCACATGGAGGGCTCCGTGAACGGCCACGAGTTCGAGATCGAGGGCGAGGGCGAGGGCCGCCCCTACGAGGGCACCCAGACCGCCAAGCTGAAGGTGACCAAGGGTGGCCCCCTGCCCTTCGCCTGGGACATCCTGTCCCCTCAGTTCATGTACGGCTCCAAGGCCTACGTGAAGCACCCCGCCGACATCCCCGACTACTTGAAGCTGTCCTTCCCCGAGGGCTTCAAGTGGGAGCGCGTGATGAACTTCGAGGACGGCGGCGTGGTGACCGTGACCCAGGACTCCTCCCTGCAGGACGGCGAGTTCATCTACAAGGTGAAGCTGCGCGGCACCAACTTCCCCTCCGACGGCCCCGTAATGCAGAAGAAGACCATGGGCTGGGAGGCCTCCTCCGAGCGGATGTACCCCGAGGACGGCGCCCTGAAGGGCGAGATCAAGCAGAGGCTGAAGCTGAAGGACGGCGGCCACTACGACGCTGAGGTCAAGACCACCTACAAGGCCAAGAAGCCCGTGCAGCTGCCCGGCGCCTACAACGTCAACATCAAGTTGGACATCACCTCCCACAACGAGGACTACACCATCGTGGAACAGTACGAACGCGCCGAGGGCCGCCACTCCACCGGCGGCATGGACGAGCTGTACAAG**TAA

**Human Long-mTq2 (hSLC26A4_long_isoform-linker-mTq2, used in Figs.4 and 5)**

**ATGGCAGCGCCAGGCGGCAGGTCGGAGCCGCCGCAGCTCCCCGAGTACAGCTGCAGCTACATGGTGTCGCGGCCGGTCTACAGCGAGCTCGCTTTCCAGCAACAGCACGAGCGGCGCCTGCAGGAGCGCAAGACGCTGCGGGAGAGCCTGGCCAAGTGCTGCAGTTGTTCAAGAAAGAGAGCCTTTGGTGTGCTAAAGACTCTTGTGCCCATCTTGGAGTGGCTCCCCAAATACCGAGTCAAGGAATGGCTGCTTAGTGACGTCATTTCGGGAGTTAGTACTGGGCTAGTGGCCACGCTGCAAGGGATGGCATATGCCCTACTAGCTGCAGTTCCTGTCGGATATGGTCTCTACTCTGCTTTTTTCCCTATCCTGACATACTTTATCTTTGGAACATCAAGACATATCTCAGTTGGACCTTTTCCAGTGGTGAGTTTAATGGTGGGATCTGTTGTTCTGAGCATGGCCCCCGACGAACACTTTCTCGTATCCAGCAGCAATGGAACTGTATTAAATACTACTATGATAGACACTGCAGCTAGAGATACAGCTAGAGTCCTGATTGCCAGTGCCCTGACTCTGCTGGTTGGAATTATACAGTTGATATTTGGTGGCTTGCAGATTGGATTCATAGTGAGGTACTTGGCAGATCCTTTGGTTGGTGGCTTCACAACAGCTGCTGCCTTCCAAGTGCTGGTCTCACAGCTAAAGATTGTCCTCAATGTTTCAACCAAAAACTACAATGGAGTTCTCTCTATTATCTATACGCTGGTTGAGATTTTTCAAAATATTGGTGATACCAATCTTGCTGATTTCACTGCTGGATTGCTCACCATTGTCGTCTGTATGGCAGTTAAGGAATTAAATGATCGGTTTAGACACAAAATCCCAGTCCCTATTCCTATAGAAGTAATTGTGACGATAATTGCTACTGCCATTTCATATGGAGCCAACCTGGAAAAAAATTACAATGCTGGCATTGTTAAATCCATCCCAAGGGGGTTTTTGCCTCCTGAACTTCCACCTGTGAGCTTGTTCTCGGAGATGCTGGCTGCATCATTTTCCATCGCTGTGGTGGCTTATGCTATTGCAGTGTCAGTAGGAAAAGTATATGCCACCAAGTATGATTACACCATCGATGGGAACCAGGAATTCATTGCCTTTGGGATCAGCAACATCTTCTCAGGATTCTTCTCTTGTTTTGTGGCCACCACTGCTCTTTCCCGCACGGCCGTCCAGGAGAGCACTGGAGGAAAGACACAGGTTGCTGGCATCATCTCTGCTGCGATTGTGATGATCGCCATTCTTGCCCTGGGGAAGCTTCTGGAACCCTTGCAGAAGTCGGTCTTGGCAGCTGTTGTAATTGCCAACCTGAAAGGGATGTTTATGCAGCTGTGTGACATTCCTCGTCTGTGGAGACAGAATAAGATTGATGCTGTTATCTGGGTGTTTACGTGTATAGTGTCCATCATTCTGGGGCTGGATCTCGGTTTACTAGCTGGCCTTATATTTGGACTGTTGACTGTGGTCCTGAGAGTTCAGTTTCCTTCTTGGAATGGCCTTGGAAGCATCCCTAGCACAGATATCTACAAAAGTACCAAGAATTACAAAAACATTGAAGAACCTCAAGGAGTGAAGATTCTTAGATTTTCCAGTCCTATTTTCTATGGCAATGTCGATGGTTTTAAAAAATGTATCAAGTCCACAGTTGGATTTGATGCCATTAGAGTATATAATAAGAGGCTGAAAGCGCTGAGGAAAATACAGAAACTAATAAAAAGTGGACAATTAAGAGCAACAAAGAATGGCATCATAAGTGATGCTGTTTCAACAAATAATGCTTTTGAGCCTGATGAGGATATTGAAGATCTGGAGGAACTTGATATCCCAACCAAGGAAATAGAGATTCAAGTGGATTGGAACTCTGAGCTTCCAGTCAAAGTGAACGTTCCCAAAGTGCCAATCCATAGCCTTGTGCTTGACTGTGGAGCTATATCTTTCCTGGACGTTGTTGGAGTGAGATCACTGCGGGTGATTGTCAAAGAATTCCAAAGAATTGATGTGAATGTGTATTTTGCATCACTTCAAGATTATGTGATAGAAAAGCTGGAGCAATGCGGGTTCTTTGACGACAACATTAGAAAGGACACATTCTTTTTGACGGTCCATGATGCTATACTCTATCTACAGAACCAAGTGAAATCTCAAGAGGGTCAAGGTTCCATTTTAGAAACGATCACTCTCATTCAGGATTGTAAAGATACCCTTGAATTAATAGAAACAGAGCTGACGGAAGAAGAACTTGATGTCCAGGATGAGGCTATGCGTACACTTGCATCCCGGGACCCACCGGTCGCCACCATGGTGAGCAAGGGCGAGGAGCTGTTCACCGGGGTGGTGCCCATCCTGGTCGAGCTGGACGGCGACGTAAACGGCCACAAGTTCAGCGTGTCCGGCGAGGGCGAGGGCGATGCCACCTACGGCAAGCTGACCCTGAAGTTCATCTGCACCACCGGCAAGCTGCCCGTGCCCTGGCCCACCCTCGTGACCACCCTGTCCTGGGGCGTGCAGTGCTTCGCCCGCTACCCCGACCACATGAAGCAGCACGACTTCTTCAAGTCCGCCATGCCCGAAGGCTACGTCCAGGAGCGCACCATCTTCTTCAAGGACGACGGCAACTACAAGACCCGCGCCGAGGTGAAGTTCGAGGGCGACACCCTGGTGAACCGCATCGAGCTGAAGGGCATCGACTTCAAGGAGGACGGCAACATCCTGGGGCACAAGCTGGAGTACAACTACTTTAGCGACAACGTCTATATCACCGCCGACAAGCAGAAGAACGGCATCAAGGCCAACTTCAAGATCCGCCACAACATCGAGGACGGCGGCGTGCAGCTCGCCGACCACTACCAGCAGAACACCCCCATCGGCGACGGCCCCGTGCTGCTGCCCGACAACCACTACCTGAGCACCCAGTCCAAGCTGAGCAAAGACCCCAACGAGAAGCGCGATCACATGGTCCTGCTGGAGTTCGTGACCGCCGCCGGGATCACTCTCGGCATGGACGAGCTGTACAAG**TAA

**Human Short-mTq2 (hSLC26A4_short_isoform-linker-mTq2, used in Figs.4 and 5)**

**ATGATCGCCATTCTTGCCCTGGGGAAGCTTCTGGAACCCTTGCAGAAGTCGGTCTTGGCAGCTGTTGTAATTGCCAACCTGAAAGGGATGTTTATGCAGCTGTGTGACATTCCTCGTCTGTGGAGACAGAATAAGATTGATGCTGTTATCTGGGTGTTTACGTGTATAGTGTCCATCATTCTGGGGCTGGATCTCGGTTTACTAGCTGGCCTTATATTTGGACTGTTGACTGTGGTCCTGAGAGTTCAGTTTCCTTCTTGGAATGGCCTTGGAAGCATCCCTAGCACAGATATCTACAAAAGTACCAAGAATTACAAAAACATTGAAGAACCTCAAGGAGTGAAGATTCTTAGATTTTCCAGTCCTATTTTCTATGGCAATGTCGATGGTTTTAAAAAATGTATCAAGTCCACAGTTGGATTTGATGCCATTAGAGTATATAATAAGAGGCTGAAAGCGCTGAGGAAAATACAGAAACTAATAAAAAGTGGACAATTAAGAGCAACAAAGAATGGCATCATAAGTGATGCTGTTTCAACAAATAATGCTTTTGAGCCTGATGAGGATATTGAAGATCTGGAGGAACTTGATATCCCAACCAAGGAAATAGAGATTCAAGTGGATTGGAACTCTGAGCTTCCAGTCAAAGTGAACGTTCCCAAAGTGCCAATCCATAGCCTTGTGCTTGACTGTGGAGCTATATCTTTCCTGGACGTTGTTGGAGTGAGATCACTGCGGGTGATTGTCAAAGAATTCCAAAGAATTGATGTGAATGTGTATTTTGCATCACTTCAAGATTATGTGATAGAAAAGCTGGAGCAATGCGGGTTCTTTGACGACAACATTAGAAAGGACACATTCTTTTTGACGGTCCATGATGCTATACTCTATCTACAGAACCAAGTGAAATCTCAAGAGGGTCAAGGTTCCATTTTAGAAACGATCACTCTCATTCAGGATTGTAAAGATACCCTTGAATTAATAGAAACAGAGCTGACGGAAGAAGAACTTGATGTCCAGGATGAGGCTATGCGTACACTTGCATCCCGGGACCCACCGGTCGCCACCATGGTGAGCAAGGGCGAGGAGCTGTTCACCGGGGTGGTGCCCATCCTGGTCGAGCTGGACGGCGACGTAAACGGCCACAAGTTCAGCGTGTCCGGCGAGGGCGAGGGCGATGCCACCTACGGCAAGCTGACCCTGAAGTTCATCTGCACCACCGGCAAGCTGCCCGTGCCCTGGCCCACCCTCGTGACCACCCTGTCCTGGGGCGTGCAGTGCTTCGCCCGCTACCCCGACCACATGAAGCAGCACGACTTCTTCAAGTCCGCCATGCCCGAAGGCTACGTCCAGGAGCGCACCATCTTCTTCAAGGACGACGGCAACTACAAGACCCGCGCCGAGGTGAAGTTCGAGGGCGACACCCTGGTGAACCGCATCGAGCTGAAGGGCATCGACTTCAAGGAGGACGGCAACATCCTGGGGCACAAGCTGGAGTACAACTACTTTAGCGACAACGTCTATATCACCGCCGACAAGCAGAAGAACGGCATCAAGGCCAACTTCAAGATCCGCCACAACATCGAGGACGGCGGCGTGCAGCTCGCCGACCACTACCAGCAGAACACCCCCATCGGCGACGGCCCCGTGCTGCTGCCCGACAACCACTACCTGAGCACCCAGTCCAAGCTGAGCAAAGACCCCAACGAGAAGCGCGATCACATGGTCCTGCTGGAGTTCGTGACCGCCGCCGGGATCACTCTCGGCATGGACGAGCTGTACAAG**TAA

**Human mTq2-P2A-Long (mTq2-P2A-hSLC26A4_long_isoform, used in Figs.5 and S7)**

**ATGGTGAGCAAGGGCGAGGAGCTGTTCACCGGGGTGGTGCCCATCCTGGTCGAGCTGGACGGCGACGTAAACGGCCACAAGTTCAGCGTGTCCGGCGAGGGCGAGGGCGATGCCACCTACGGCAAGCTGACCCTGAAGTTCATCTGCACCACCGGCAAGCTGCCCGTGCCCTGGCCCACCCTCGTGACCACCCTGTCCTGGGGCGTGCAGTGCTTCGCCCGCTACCCCGACCACATGAAGCAGCACGACTTCTTCAAGTCCGCCATGCCCGAAGGCTACGTCCAGGAGCGCACCATCTTCTTCAAGGACGACGGCAACTACAAGACCCGCGCCGAGGTGAAGTTCGAGGGCGACACCCTGGTGAACCGCATCGAGCTGAAGGGCATCGACTTCAAGGAGGACGGCAACATCCTGGGGCACAAGCTGGAGTACAACTACTTTAGCGACAACGTCTATATCACCGCCGACAAGCAGAAGAACGGCATCAAGGCCAACTTCAAGATCCGCCACAACATCGAGGACGGCGGCGTGCAGCTCGCCGACCACTACCAGCAGAACACCCCCATCGGCGACGGCCCCGTGCTGCTGCCCGACAACCACTACCTGAGCACCCAGTCCAAGCTGAGCAAAGACCCCAACGAGAAGCGCGATCACATGGTCCTGCTGGAGTTCGTGACCGCCGCCGGGATCACTCTCGGCATGGACGAGCTGTACAAGGGAAGCGGAGCTACTAACTTCAGCCTGCTGAAGCAGGCTGGAGACGTCGAGGAGAACCCTGGACCTATGGCAGCGCCAGGCGGCAGGTCGGAGCCGCCGCAGCTCCCCGAGTACAGCTGCAGCTACATGGTGTCGCGGCCGGTCTACAGCGAGCTCGCTTTCCAGCAACAGCACGAGCGGCGCCTGCAGGAGCGCAAGACGCTGCGGGAGAGCCTGGCCAAGTGCTGCAGTTGTTCAAGAAAGAGAGCCTTTGGTGTGCTAAAGACTCTTGTGCCCATCTTGGAGTGGCTCCCCAAATACCGAGTCAAGGAATGGCTGCTTAGTGACGTCATTTCGGGAGTTAGTACTGGGCTAGTGGCCACGCTGCAAGGGATGGCATATGCCCTACTAGCTGCAGTTCCTGTCGGATATGGTCTCTACTCTGCTTTTTTCCCTATCCTGACATACTTTATCTTTGGAACATCAAGACATATCTCAGTTGGACCTTTTCCAGTGGTGAGTTTAATGGTGGGATCTGTTGTTCTGAGCATGGCCCCCGACGAACACTTTCTCGTATCCAGCAGCAATGGAACTGTATTAAATACTACTATGATAGACACTGCAGCTAGAGATACAGCTAGAGTCCTGATTGCCAGTGCCCTGACTCTGCTGGTTGGAATTATACAGTTGATATTTGGTGGCTTGCAGATTGGATTCATAGTGAGGTACTTGGCAGATCCTTTGGTTGGTGGCTTCACAACAGCTGCTGCCTTCCAAGTGCTGGTCTCACAGCTAAAGATTGTCCTCAATGTTTCAACCAAAAACTACAATGGAGTTCTCTCTATTATCTATACGCTGGTTGAGATTTTTCAAAATATTGGTGATACCAATCTTGCTGATTTCACTGCTGGATTGCTCACCATTGTCGTCTGTATGGCAGTTAAGGAATTAAATGATCGGTTTAGACACAAAATCCCAGTCCCTATTCCTATAGAAGTAATTGTGACGATAATTGCTACTGCCATTTCATATGGAGCCAACCTGGAAAAAAATTACAATGCTGGCATTGTTAAATCCATCCCAAGGGGGTTTTTGCCTCCTGAACTTCCACCTGTGAGCTTGTTCTCGGAGATGCTGGCTGCATCATTTTCCATCGCTGTGGTGGCTTATGCTATTGCAGTGTCAGTAGGAAAAGTATATGCCACCAAGTATGATTACACCATCGATGGGAACCAGGAATTCATTGCCTTTGGGATCAGCAACATCTTCTCAGGATTCTTCTCTTGTTTTGTGGCCACCACTGCTCTTTCCCGCACGGCCGTCCAGGAGAGCACTGGAGGAAAGACACAGGTTGCTGGCATCATCTCTGCTGCGATTGTGATGATCGCCATTCTTGCCCTGGGGAAGCTTCTGGAACCCTTGCAGAAGTCGGTCTTGGCAGCTGTTGTAATTGCCAACCTGAAAGGGATGTTTATGCAGCTGTGTGACATTCCTCGTCTGTGGAGACAGAATAAGATTGATGCTGTTATCTGGGTGTTTACGTGTATAGTGTCCATCATTCTGGGGCTGGATCTCGGTTTACTAGCTGGCCTTATATTTGGACTGTTGACTGTGGTCCTGAGAGTTCAGTTTCCTTCTTGGAATGGCCTTGGAAGCATCCCTAGCACAGATATCTACAAAAGTACCAAGAATTACAAAAACATTGAAGAACCTCAAGGAGTGAAGATTCTTAGATTTTCCAGTCCTATTTTCTATGGCAATGTCGATGGTTTTAAAAAATGTATCAAGTCCACAGTTGGATTTGATGCCATTAGAGTATATAATAAGAGGCTGAAAGCGCTGAGGAAAATACAGAAACTAATAAAAAGTGGACAATTAAGAGCAACAAAGAATGGCATCATAAGTGATGCTGTTTCAACAAATAATGCTTTTGAGCCTGATGAGGATATTGAAGATCTGGAGGAACTTGATATCCCAACCAAGGAAATAGAGATTCAAGTGGATTGGAACTCTGAGCTTCCAGTCAAAGTGAACGTTCCCAAAGTGCCAATCCATAGCCTTGTGCTTGACTGTGGAGCTATATCTTTCCTGGACGTTGTTGGAGTGAGATCACTGCGGGTGATTGTCAAAGAATTCCAAAGAATTGATGTGAATGTGTATTTTGCATCACTTCAAGATTATGTGATAGAAAAGCTGGAGCAATGCGGGTTCTTTGACGACAACATTAGAAAGGACACATTCTTTTTGACGGTCCATGATGCTATACTCTATCTACAGAACCAAGTGAAATCTCAAGAGGGTCAAGGTTCCATTTTAGAAACGATCACTCTCATTCAGGATTGTAAAGATACCCTTGAATTAATAGAAACAGAGCTGACGGAAGAAGAACTTGATGTCCAGGATGAGGCTATGCGTACACTTGCATCC**TGA

**Human mTq2-P2A-Short (mTq2-P2A-hSLC26A4_short_isoform, used in Figs.5 and S7)**

**ATGGTGAGCAAGGGCGAGGAGCTGTTCACCGGGGTGGTGCCCATCCTGGTCGAGCTGGACGGCGACGTAAACGGCCACAAGTTCAGCGTGTCCGGCGAGGGCGAGGGCGATGCCACCTACGGCAAGCTGACCCTGAAGTTCATCTGCACCACCGGCAAGCTGCCCGTGCCCTGGCCCACCCTCGTGACCACCCTGTCCTGGGGCGTGCAGTGCTTCGCCCGCTACCCCGACCACATGAAGCAGCACGACTTCTTCAAGTCCGCCATGCCCGAAGGCTACGTCCAGGAGCGCACCATCTTCTTCAAGGACGACGGCAACTACAAGACCCGCGCCGAGGTGAAGTTCGAGGGCGACACCCTGGTGAACCGCATCGAGCTGAAGGGCATCGACTTCAAGGAGGACGGCAACATCCTGGGGCACAAGCTGGAGTACAACTACTTTAGCGACAACGTCTATATCACCGCCGACAAGCAGAAGAACGGCATCAAGGCCAACTTCAAGATCCGCCACAACATCGAGGACGGCGGCGTGCAGCTCGCCGACCACTACCAGCAGAACACCCCCATCGGCGACGGCCCCGTGCTGCTGCCCGACAACCACTACCTGAGCACCCAGTCCAAGCTGAGCAAAGACCCCAACGAGAAGCGCGATCACATGGTCCTGCTGGAGTTCGTGACCGCCGCCGGGATCACTCTCGGCATGGACGAGCTGTACAAGGGAAGCGGAGCTACTAACTTCAGCCTGCTGAAGCAGGCTGGAGACGTCGAGGAGAACCCTGGACCTATGATCGCCATTCTTGCCCTGGGGAAGCTTCTGGAACCCTTGCAGAAGTCGGTCTTGGCAGCTGTTGTAATTGCCAACCTGAAAGGGATGTTTATGCAGCTGTGTGACATTCCTCGTCTGTGGAGACAGAATAAGATTGATGCTGTTATCTGGGTGTTTACGTGTATAGTGTCCATCATTCTGGGGCTGGATCTCGGTTTACTAGCTGGCCTTATATTTGGACTGTTGACTGTGGTCCTGAGAGTTCAGTTTCCTTCTTGGAATGGCCTTGGAAGCATCCCTAGCACAGATATCTACAAAAGTACCAAGAATTACAAAAACATTGAAGAACCTCAAGGAGTGAAGATTCTTAGATTTTCCAGTCCTATTTTCTATGGCAATGTCGATGGTTTTAAAAAATGTATCAAGTCCACAGTTGGATTTGATGCCATTAGAGTATATAATAAGAGGCTGAAAGCGCTGAGGAAAATACAGAAACTAATAAAAAGTGGACAATTAAGAGCAACAAAGAATGGCATCATAAGTGATGCTGTTTCAACAAATAATGCTTTTGAGCCTGATGAGGATATTGAAGATCTGGAGGAACTTGATATCCCAACCAAGGAAATAGAGATTCAAGTGGATTGGAACTCTGAGCTTCCAGTCAAAGTGAACGTTCCCAAAGTGCCAATCCATAGCCTTGTGCTTGACTGTGGAGCTATATCTTTCCTGGACGTTGTTGGAGTGAGATCACTGCGGGTGATTGTCAAAGAATTCCAAAGAATTGATGTGAATGTGTATTTTGCATCACTTCAAGATTATGTGATAGAAAAGCTGGAGCAATGCGGGTTCTTTGACGACAACATTAGAAAGGACACATTCTTTTTGACGGTCCATGATGCTATACTCTATCTACAGAACCAAGTGAAATCTCAAGAGGGTCAAGGTTCCATTTTAGAAACGATCACTCTCATTCAGGATTGTAAAGATACCCTTGAATTAATAGAAACAGAGCTGACGGAAGAAGAACTTGATGTCCAGGATGAGGCTATGCGTACACTTGCATCC**

**Human miRFP670-P2A-Long (miRFP670-P2A-hSLC26A4_long_isoform used in Figs.5 and S7)**

**ATGGTAGCAGGTCATGCCTCTGGCAGCCCCGCATTCGGGACCGCCTCTCATTCGAATTGCGAACATGAAGAGATCCACCTCGCCGGCTCGATCCAGCCGCATGGCGCGCTTCTGGTCGTCAGCGAACATGATCATCGCGTCATCCAGGCCAGCGCCAACGCCGCGGAATTTCTGAATCTCGGAAGCGTACTCGGCGTTCCGCTCGCCGAGATCGACGGCGATCTGTTGATCAAGATCCTGCCGCATCTCGATCCCACCGCCGAAGGCATGCCGGTCGCGGTGCGCTGCCGGATCGGCAATCCCTCTACGGAGTACTGCGGTCTGATGCATCGGCCTCCGGAAGGCGGGCTGATCATCGAACTCGAACGTGCCGGCCCGTCGATCGATCTGTCAGGCACGCTGGCGCCGGCGCTGGAGCGGATCCGCACGGCGGGTTCACTGCGCGCGCTGTGCGATGACACCGTGCTGCTGTTTCAGCAGTGCACCGGCTACGACCGGGTGATGGTGTATCGTTTCGATGAGCAAGGCCACGGCCTGGTATTCTCCGAGTGCCATGTGCCTGGGCTCGAATCCTATTTCGGCAACCGCTATCCGTCGTCGACTGTCCCGCAGATGGCGCGGCAGCTGTACGTGCGGCAGCGCGTCCGCGTGCTGGTCGACGTCACCTATCAGCCGGTGCCGCTGGAGCCGCGGCTGTCGCCGCTGACCGGGCGCGATCTCGACATGTCGGGCTGCTTCCTGCGCTCGATGTCGCCGTGCCATCTGCAGTTCCTGAAGGACATGGGCGTGCGCGCCACCCTGGCGGTGTCGCTGGTGGTCGGCGGCAAGCTGTGGGGCCTGGTTGTCTGTCACCATTATCTGCCGCGCTTCATCCGTTTCGAGCTGCGGGCGATCTGCAAACGGCTCGCCGAAAGGATCGCGACGCGGATCACCGCGCTTGAGAGCGGAAGCGGAGCTACTAACTTCAGCCTGCTGAAGCAGGCTGGAGACGTCGAGGAGAACCCTGGACCTATGGCAGCGCCAGGCGGCAGGTCGGAGCCGCCGCAGCTCCCCGAGTACAGCTGCAGCTACATGGTGTCGCGGCCGGTCTACAGCGAGCTCGCTTTCCAGCAACAGCACGAGCGGCGCCTGCAGGAGCGCAAGACGCTGCGGGAGAGCCTGGCCAAGTGCTGCAGTTGTTCAAGAAAGAGAGCCTTTGGTGTGCTAAAGACTCTTGTGCCCATCTTGGAGTGGCTCCCCAAATACCGAGTCAAGGAATGGCTGCTTAGTGACGTCATTTCGGGAGTTAGTACTGGGCTAGTGGCCACGCTGCAAGGGATGGCATATGCCCTACTAGCTGCAGTTCCTGTCGGATATGGTCTCTACTCTGCTTTTTTCCCTATCCTGACATACTTTATCTTTGGAACATCAAGACATATCTCAGTTGGACCTTTTCCAGTGGTGAGTTTAATGGTGGGATCTGTTGTTCTGAGCATGGCCCCCGACGAACACTTTCTCGTATCCAGCAGCAATGGAACTGTATTAAATACTACTATGATAGACACTGCAGCTAGAGATACAGCTAGAGTCCTGATTGCCAGTGCCCTGACTCTGCTGGTTGGAATTATACAGTTGATATTTGGTGGCTTGCAGATTGGATTCATAGTGAGGTACTTGGCAGATCCTTTGGTTGGTGGCTTCACAACAGCTGCTGCCTTCCAAGTGCTGGTCTCACAGCTAAAGATTGTCCTCAATGTTTCAACCAAAAACTACAATGGAGTTCTCTCTATTATCTATACGCTGGTTGAGATTTTTCAAAATATTGGTGATACCAATCTTGCTGATTTCACTGCTGGATTGCTCACCATTGTCGTCTGTATGGCAGTTAAGGAATTAAATGATCGGTTTAGACACAAAATCCCAGTCCCTATTCCTATAGAAGTAATTGTGACGATAATTGCTACTGCCATTTCATATGGAGCCAACCTGGAAAAAAATTACAATGCTGGCATTGTTAAATCCATCCCAAGGGGGTTTTTGCCTCCTGAACTTCCACCTGTGAGCTTGTTCTCGGAGATGCTGGCTGCATCATTTTCCATCGCTGTGGTGGCTTATGCTATTGCAGTGTCAGTAGGAAAAGTATATGCCACCAAGTATGATTACACCATCGATGGGAACCAGGAATTCATTGCCTTTGGGATCAGCAACATCTTCTCAGGATTCTTCTCTTGTTTTGTGGCCACCACTGCTCTTTCCCGCACGGCCGTCCAGGAGAGCACTGGAGGAAAGACACAGGTTGCTGGCATCATCTCTGCTGCGATTGTGATGATCGCCATTCTTGCCCTGGGGAAGCTTCTGGAACCCTTGCAGAAGTCGGTCTTGGCAGCTGTTGTAATTGCCAACCTGAAAGGGATGTTTATGCAGCTGTGTGACATTCCTCGTCTGTGGAGACAGAATAAGATTGATGCTGTTATCTGGGTGTTTACGTGTATAGTGTCCATCATTCTGGGGCTGGATCTCGGTTTACTAGCTGGCCTTATATTTGGACTGTTGACTGTGGTCCTGAGAGTTCAGTTTCCTTCTTGGAATGGCCTTGGAAGCATCCCTAGCACAGATATCTACAAAAGTACCAAGAATTACAAAAACATTGAAGAACCTCAAGGAGTGAAGATTCTTAGATTTTCCAGTCCTATTTTCTATGGCAATGTCGATGGTTTTAAAAAATGTATCAAGTCCACAGTTGGATTTGATGCCATTAGAGTATATAATAAGAGGCTGAAAGCGCTGAGGAAAATACAGAAACTAATAAAAAGTGGACAATTAAGAGCAACAAAGAATGGCATCATAAGTGATGCTGTTTCAACAAATAATGCTTTTGAGCCTGATGAGGATATTGAAGATCTGGAGGAACTTGATATCCCAACCAAGGAAATAGAGATTCAAGTGGATTGGAACTCTGAGCTTCCAGTCAAAGTGAACGTTCCCAAAGTGCCAATCCATAGCCTTGTGCTTGACTGTGGAGCTATATCTTTCCTGGACGTTGTTGGAGTGAGATCACTGCGGGTGATTGTCAAAGAATTCCAAAGAATTGATGTGAATGTGTATTTTGCATCACTTCAAGATTATGTGATAGAAAAGCTGGAGCAATGCGGGTTCTTTGACGACAACATTAGAAAGGACACATTCTTTTTGACGGTCCATGATGCTATACTCTATCTACAGAACCAAGTGAAATCTCAAGAGGGTCAAGGTTCCATTTTAGAAACGATCACTCTCATTCAGGATTGTAAAGATACCCTTGAATTAATAGAAACAGAGCTGACGGAAGAAGAACTTGATGTCCAGGATGAGGCTATGCGTACACTTGCATCC**TGA

**Mouse Long-mTq2 (mSLC26A4_long_isoform-linker-mTq2, used in Fig.S7)**

**ATGGCAGCGCGGGGCGGCAGGTCGGAGCCGCCGCAGCTCGCCGAGTACAGCTGCAGTTACACGGTATCGCGGCCGGTGTACAGCGAGCTCGCCTTCCAGCAGCAGCGCGAGCGGCGCCTGCCTGAGCGCAGGACGCTGCGGGACAGCCTGGCGCGGAGCTGCAGTTGCTCAAGAAAGAGAGCCTTTGGTGTGGTAAAGACTCTCCTGCCCATTCTGGACTGGCTCCCAAAATACCGAGTCAAGGAATGGCTCCTCAGTGACATCATCTCCGGAGTTAGCACTGGGCTGGTGGGTACCCTGCAAGGGATGGCTTATGCCCTGCTGGCAGCAGTACCTGTTCAGTTCGGTCTCTACTCTGCCTTTTTCCCTATCCTGACGTATTTTGTGTTCGGAACATCAAGACACATCTCAGTTGGCCCTTTCCCCGTGGTCAGTTTAATGGTGGGATCTGTTGTTCTGAGCATGGCTCCAGATGACCACTTTCTTGTGCCCAGCGGTAACAGAAGTGCATTGAACTCGACCACGTTAGACACTGGAACCAGAGATGCGGCCCGAGTGTTGCTTGCAAGCACACTCACTCTTCTAGTTGGAATCATACAGCTGGTGTTTGGAGGTTTGCAGATTGGATTCATAGTGAGGTACTTGGCAGACCCCTTGGTTGGCGGATTCACAACCGCTGCAGCCTTCCAAGTACTGGTCTCACAGCTAAAGATCGTGCTCAATGTTTCAACCAAAAACTACAACGGCATCCTCTCCATTATCTACACACTAATTGAGATTTTTCAAAATATCGGTGACACCAATATTGCCGATTTCATCGCTGGGCTGCTGACCATCATCGTCTGTATGGCTGTTAAGGAACTAAATGATCGATTTAAACACAGAATCCCGGTGCCCATTCCTATAGAAGTGATTGTGACAATAATTGCTACTGCCATTTCCTATGGGGCCAACTTGGAAAAGAACTACAATGCTGGCATTGTTAAGTCCATCCCAAGTGGGTTCTTGCCTCCTGTCCTGCCATCTGTGGGCCTGTTTTCGGACATGTTGGCTGCATCCTTTTCCATTGCTGTGGTGGCTTACGCTATTGCAGTGTCTGTAGGAAAAGTCTACGCCACTAAGCATGACTATGTCATCGATGGGAACCAGGAATTCATTGCCTTTGGGATAAGCAACGTCTTCTCTGGATTTTTCTCCTGTTTTGTGGCTACCACTGCTCTGTCTCGAACGGCTGTCCAGGAGAGCACCGGGGGGAAGACACAGGTGGCTGGCCTCATCTCAGCTGTGATTGTGATGGTTGCCATCGTTGCCTTGGGGAGGCTTCTGGAACCCTTGCAGAAGTCAGTCTTGGCGGCCGTTGTCATTGCCAACCTGAAAGGGATGTTTATGCAGGTGTGTGACGTTCCTCGTCTGTGGAAGCAGAATAAGACTGATGCTGTTATCTGGGTGTTTACATGCATAATGTCCATCATTCTGGGGCTGGACCTCGGCTTGCTAGCTGGCCTTTTATTTGCACTACTGACTGTGGTCCTGAGAGTTCAGTTCCCTTCATGGAATGGCCTTGGAAGTGTCCCCAGCACAGACATCTACAAAAGCATCACACATTATAAAAACCTTGAAGAGCCTGAAGGGGTGAAGATCCTGAGATTTTCCAGTCCCATTTTTTACGGCAATGTCGATGGTTTTAAAAAATGTATCAATTCAACGGTTGGATTTGATGCCATTAGAGTATATAATAAGAGGCTGAAAGCACTGAGAAGAATACAGAAACTCATCAAAAAAGGACAACTCAGGGCAACCAAGAACGGGATCATAAGTGATATTGGCTCATCAAATAATGCCTTCGAGCCTGATGAAGATGTGGAAGAGCCAGAGGAACTTAATATCCCAACCAAAGAAATTGAGATTCAAGTGGACTGGAACTCCGAACTCCCGGTGAAAGTGAATGTCCCGAAGGTGCCAATCCACAGCCTGGTGCTGGATTGCGGAGCTGTATCCTTCCTGGATGTGGTAGGAGTGAGGTCATTGCGAATGATTGTCAAAGAATTTCAGAGAATTGATGTGAATGTGTACTTTGCTCTGCTTCAAGATGATGTGTTAGAAAAGATGGAGCAGTGTGGGTTCTTCGATGACAACATTAGAAAGGACAGATTCTTTCTGACGGTTCATGATGCAATCCTCCATCTGCAGAACCAGGTCAAATCCAGAGAAGGCCAGGATTCCCTGCTAGAGACGGTCGCTCGCATTCGGGACTGTAAAGACCCTCTTGATCTGATGGAGGCAGAGATGAATGCAGAAGAGCTCGATGTTCAGGATGAGGCCATGCGTAGACTTGCTTCCCGGGACCCACCGGTCGCCACCATGGTGAGCAAGGGCGAGGAGCTGTTCACCGGGGTGGTGCCCATCCTGGTCGAGCTGGACGGCGACGTAAACGGCCACAAGTTCAGCGTGTCCGGCGAGGGCGAGGGCGATGCCACCTACGGCAAGCTGACCCTGAAGTTCATCTGCACCACCGGCAAGCTGCCCGTGCCCTGGCCCACCCTCGTGACCACCCTGTCCTGGGGCGTGCAGTGCTTCGCCCGCTACCCCGACCACATGAAGCAGCACGACTTCTTCAAGTCCGCCATGCCCGAAGGCTACGTCCAGGAGCGCACCATCTTCTTCAAGGACGACGGCAACTACAAGACCCGCGCCGAGGTGAAGTTCGAGGGCGACACCCTGGTGAACCGCATCGAGCTGAAGGGCATCGACTTCAAGGAGGACGGCAACATCCTGGGGCACAAGCTGGAGTACAACTACTTTAGCGACAACGTCTATATCACCGCCGACAAGCAGAAGAACGGCATCAAGGCCAACTTCAAGATCCGCCACAACATCGAGGACGGCGGCGTGCAGCTCGCCGACCACTACCAGCAGAACACCCCCATCGGCGACGGCCCCGTGCTGCTGCCCGACAACCACTACCTGAGCACCCAGTCCAAGCTGAGCAAAGACCCCAACGAGAAGCGCGATCACATGGTCCTGCTGGAGTTCGTGACCGCCGCCGGGATCACTCTCGGCATGGACGAGCTGTACAAG**TAA

**Mouse Short-mTq2 (mSLC26A4_short_isoform-linker-mTq2, used in Fig.S7)**

**ATGGTTGCCATCGTTGCCTTGGGGAGGCTTCTGGAACCCTTGCAGAAGTCAGTCTTGGCGGCCGTTGTCATTGCCAACCTGAAAGGGATGTTTATGCAGGTGTGTGACGTTCCTCGTCTGTGGAAGCAGAATAAGACTGATGCTGTTATCTGGGTGTTTACATGCATAATGTCCATCATTCTGGGGCTGGACCTCGGCTTGCTAGCTGGCCTTTTATTTGCACTACTGACTGTGGTCCTGAGAGTTCAGTTCCCTTCATGGAATGGCCTTGGAAGTGTCCCCAGCACAGACATCTACAAAAGCATCACACATTATAAAAACCTTGAAGAGCCTGAAGGGGTGAAGATCCTGAGATTTTCCAGTCCCATTTTTTACGGCAATGTCGATGGTTTTAAAAAATGTATCAATTCAACGGTTGGATTTGATGCCATTAGAGTATATAATAAGAGGCTGAAAGCACTGAGAAGAATACAGAAACTCATCAAAAAAGGACAACTCAGGGCAACCAAGAACGGGATCATAAGTGATATTGGCTCATCAAATAATGCCTTCGAGCCTGATGAAGATGTGGAAGAGCCAGAGGAACTTAATATCCCAACCAAAGAAATTGAGATTCAAGTGGACTGGAACTCCGAACTCCCGGTGAAAGTGAATGTCCCGAAGGTGCCAATCCACAGCCTGGTGCTGGATTGCGGAGCTGTATCCTTCCTGGATGTGGTAGGAGTGAGGTCATTGCGAATGATTGTCAAAGAATTTCAGAGAATTGATGTGAATGTGTACTTTGCTCTGCTTCAAGATGATGTGTTAGAAAAGATGGAGCAGTGTGGGTTCTTCGATGACAACATTAGAAAGGACAGATTCTTTCTGACGGTTCATGATGCAATCCTCCATCTGCAGAACCAGGTCAAATCCAGAGAAGGCCAGGATTCCCTGCTAGAGACGGTCGCTCGCATTCGGGACTGTAAAGACCCTCTTGATCTGATGGAGGCAGAGATGAATGCAGAAGAGCTCGATGTTCAGGATGAGGCCATGCGTAGACTTGCTTCCCGGGACCCACCGGTCGCCACCATGGTGAGCAAGGGCGAGGAGCTGTTCACCGGGGTGGTGCCCATCCTGGTCGAGCTGGACGGCGACGTAAACGGCCACAAGTTCAGCGTGTCCGGCGAGGGCGAGGGCGATGCCACCTACGGCAAGCTGACCCTGAAGTTCATCTGCACCACCGGCAAGCTGCCCGTGCCCTGGCCCACCCTCGTGACCACCCTGTCCTGGGGCGTGCAGTGCTTCGCCCGCTACCCCGACCACATGAAGCAGCACGACTTCTTCAAGTCCGCCATGCCCGAAGGCTACGTCCAGGAGCGCACCATCTTCTTCAAGGACGACGGCAACTACAAGACCCGCGCCGAGGTGAAGTTCGAGGGCGACACCCTGGTGAACCGCATCGAGCTGAAGGGCATCGACTTCAAGGAGGACGGCAACATCCTGGGGCACAAGCTGGAGTACAACTACTTTAGCGACAACGTCTATATCACCGCCGACAAGCAGAAGAACGGCATCAAGGCCAACTTCAAGATCCGCCACAACATCGAGGACGGCGGCGTGCAGCTCGCCGACCACTACCAGCAGAACACCCCCATCGGCGACGGCCCCGTGCTGCTGCCCGACAACCACTACCTGAGCACCCAGTCCAAGCTGAGCAAAGACCCCAACGAGAAGCGCGATCACATGGTCCTGCTGGAGTTCGTGACCGCCGCCGGGATCACTCTCGGCATGGACGAGCTGTACAAG**TAA
