# Supplementary material for: Identification of a novel isoform of Slc26a4 by single-cell RNA-sequencing of pendrin-expressing cells in the cochlea
Source: Hum Genet. 2026 Jul 16;145(1):62. doi: 10.1007/s00439-026-02858-x (PMC13375699; doi:10.1007/s00439-026-02858-x)
Supplement: Supplementary file 2 — Supplementary Material 2 [file 439_2026_2858_MOESM2_ESM.docx]

**Supplementary Fig. 9. R code used in this study**

library(readr)

library(ggplot2)

library(RColorBrewer)

library(gtools)

Isoform_expression_12cell <- read_csv("C:/Users/jkoh/UI/Isoform_expression_Slc26a4_12cells_mod.csv",

col_names = FALSE)

head(Isoform_expression_12cell)

Isoform_Numbers_12cell <- gsub("Isoform", "", Isoform_expression_12cell$X1)

Isoform_expression_12cell["Isoform_Numbers"] <- as.numeric(Isoform_Numbers_12cell)

rm(Isoform_Numbers_12cell)

Isoform_expression_12cell <- data.frame(Isoform_expression_12cell)

Isoform_expression_12cell_old <- data.frame(Isoform_expression_12cell)

Isoform_expression_12cell_sorted <- Isoform_expression_12cell[ order(Isoform_expression_12cell[,14],

Isoform_expression_12cell[,1]),]

rownames(Isoform_expression_12cell_sorted) <-c(Isoform_expression_12cell_sorted$Isoform_Numbers)

Isoform_expression_12cell_sorted$X1 <- NULL

Isoform_expression_12cell_sorted$Isoform_Numbers <- NULL

colnames(Isoform_expression_12cell_sorted) <-

c("Cell_02", "Cell_03", "Cell_05", "Cell_09", "Cell_10", "Cell_11",

"Cell_13", "Cell_14", "Cell_16", "Cell_18", "Cell_19", "Cell_20")

Isoform_expression_12cell <- Isoform_expression_12cell_sorted

rm(Isoform_expression_12cell_sorted)

rm(Isoform_expression_12cell_old)

str(Isoform_expression_12cell)

#####################################################

# Count the number of isoforms observations #########

#####################################################

AvgIsoformExpression_12 <- rowMeans(Isoform_expression_12cell, na.rm = FALSE)

barplot(AvgIsoformExpression_12)

# Get number of isoform occurances

IsoformOccurnaces_12 <- rowSums(Isoform_expression_12cell != 0)

barplot(IsoformOccurnaces_12)

#######################################################

#### Combine redundant isoforms #######################################################

# Combine isoforms 1,2,3 and 4 #short isoform

Isoform1 <- rbind.data.frame(Isoform_expression_12cell[1,], Isoform_expression_12cell[2,], Isoform_expression_12cell[3,], Isoform_expression_12cell[4,])

Isoform1 <- colSums(Isoform1)

Isoform1["Isoform"] <- c(1)

# Isoforms 2 #isoforms 10 and 11 #short isoform

Isoform2 <- rbind.data.frame(Isoform_expression_12cell[8,], Isoform_expression_12cell[9,])

Isoform2 <- colSums(Isoform2)

Isoform2["Isoform"] <- c(2)

# Isoforms 3 ## #isoforms 5 and 12 #short isoform ## Minor shorter isoform

Isoform3 <- rbind.data.frame(Isoform_expression_12cell[5,], Isoform_expression_12cell[10,])

Isoform3 <- colSums(Isoform3)

Isoform3["Isoform"] <- c(3)

# Isoforms 4 ## #isoforms 7,8 and 13 #short isoform

Isoform4 <- rbind.data.frame(Isoform_expression_12cell[6,], Isoform_expression_12cell[7,], Isoform_expression_12cell[11,])

Isoform4 <- colSums(Isoform4)

Isoform4["Isoform"] <- c(4)

# Create a new dataframe with consolidated redundant rows

Isoform_expression_12cell_2 <- Isoform_expression_12cell[-c(1,2,3,4,5,6,7,8,9,10),]

# Make sure the isoform labels column is numeric

Isoform_expression_12cell_2["Isoform"] <- as.numeric(rownames(Isoform_expression_12cell_2))

# test

Isoform_expression_12cell_3 <- rbind.data.frame(Isoform1, Isoform_expression_12cell_2)

# Combine data frames with the new consolidated isoform values

Isoform_expression_12cell_3 <- rbind.data.frame(Isoform1,

Isoform2,

Isoform3,

Isoform4,

Isoform_expression_12cell_2)

Isoform_expression_12cell_3 <- Isoform_expression_12cell_3[-c(5),]

# Set the rownames to match the isoform numbers

rownames(Isoform_expression_12cell_3) <- Isoform_expression_12cell_3$Isoform

# Sort the data by isoform number

Isoform_expression_12cell_3 <-

Isoform_expression_12cell_3[ order(Isoform_expression_12cell_3$Isoform),]

Isoform_expression_12cell_3$Isoform <- NULL

View(Isoform_expression_12cell_3)

class(Isoform_expression_12cell_3)

Isoform_expression_12cell_3$SUM <- rowSums(Isoform_expression_12cell_3)

sum(Isoform_expression_12cell_3$SUM)

Isoform_expression_12cell_3$Percent <-(Isoform_expression_12cell_3$SUM/sum(Isoform_expression_12cell_3$SUM))*100

barplot(Isoform_expression_12cell_3$Percent,

col = c("#6BAED6", "#EFF3FF", "#BDD7E7", "#2171B5"), ylim = c(0,50), ylab = "Percent Expression")

### Get summary stats for the Isoform Expression spreadsheet

library(matrixStats)

library(ggplot2)

Isoform_expression_12cell_4 <- Isoform_expression_12cell_3

Means <- rowMeans(Isoform_expression_12cell_4, na.rm = TRUE)

SD <- rowSds(as.matrix(Isoform_expression_12cell_4))

SEM <- SD/sqrt(10)

Isoform <- as.numeric(rownames(Isoform_expression_12cell_4))

library("RColorBrewer")

display.brewer.all()

# View a single RColorBrewer palette by specifying its name

display.brewer.pal(n = 8, name = 'RdBu')

# Hexadecimal color specification

brewer.pal(n = 8, name = "RdBu")

brewer.pal(n = 8, name = "Blues")

# Barplot using RColorBrewer

barplot(c(2,5,7), col=brewer.pal(n = 3, name = "RdBu"))

barplot(c(7,5,2), col=brewer.pal(n = 3, name = "RdBu"))

barplot(c(1,2,3,4), col=brewer.pal(n = 4, name = "RdBu"))

barplot(c(1,2,3,4), col=brewer.pal(n = 4, name = "Blues"))

ColorJYK4 <- c("#6BAED6", "#EFF3FF", "#BDD7E7", "#2171B5")

Cols4 <- as.vector(ColorJYK4)

IsoformOccurnaces <- (rowSums(Isoform_expression_12cell_3 != 0) -1)

IsoformOccurnaces <- (rowSums(Isoform_expression_12cell_3 != 0))

IsoformNumbering <- c(1:4)

### Create SummaryStats data frame used for plotting with ggplot2

SummaryStats <- data.frame(Means, SD, SEM, Isoform, Cols4,IsoformOccurnaces,

IsoformNumbering, stringsAsFactors = FALSE)

rownames(SummaryStats) <- NULL

colnames(SummaryStats) <- c("Mean", "SD", "SEM", "Isoform", "Cols", "Occ", "IsoNumbering")

# Deal with the factor misordering prolbem to obtain correctly ordered isoforms

SummaryStats$Isoform <- reorder(SummaryStats$Isoform, 1:4, order = is.ordered(SummaryStats$Isoform))

# Make a ggplot barplot with error bars for isoform expression

g2 <- ggplot(SummaryStats, aes(x=IsoformNumbering, y=Mean)) +

geom_bar(position = position_dodge(), stat = "identity", fill=Cols4) +

geom_errorbar(aes(ymin=Mean-SEM, ymax=Mean+SEM),

width=.2,

position = position_dodge(.9))

g2 + labs(x = "Isoform Number", y = "Expression values (RPG10K)")

# Make a ggplot barplot for number of occurances

p2 <- ggplot(SummaryStats, aes(x=IsoformNumbering, y=Occ)) +

geom_bar(position = position_dodge(), stat = "identity", fill=Cols4)

p2 + labs(x = "Isoform Number", y = "Cells")

# Pie chart

labels <- c("Isoform 1", "Isoform 2", "Isoform 3", "Isoform 4")

piepercent02 <- round(100*Isoform_expression_12cell_3$Cell_02/sum(Isoform_expression_12cell_3$Cell_02), 1)

piepercent03 <- round(100*Isoform_expression_12cell_3$Cell_03/sum(Isoform_expression_12cell_3$Cell_03), 1)

piepercent05 <- round(100*Isoform_expression_12cell_3$Cell_05/sum(Isoform_expression_12cell_3$Cell_05), 1)

piepercent09 <- round(100*Isoform_expression_12cell_3$Cell_09/sum(Isoform_expression_12cell_3$Cell_09), 1)

piepercent10 <- round(100*Isoform_expression_12cell_3$Cell_10/sum(Isoform_expression_12cell_3$Cell_10), 1)

piepercent11 <- round(100*Isoform_expression_12cell_3$Cell_11/sum(Isoform_expression_12cell_3$Cell_11), 1)

piepercent13 <- round(100*Isoform_expression_12cell_3$Cell_13/sum(Isoform_expression_12cell_3$Cell_13), 1)

piepercent14 <- round(100*Isoform_expression_12cell_3$Cell_14/sum(Isoform_expression_12cell_3$Cell_14), 1)

piepercent16 <- round(100*Isoform_expression_12cell_3$Cell_16/sum(Isoform_expression_12cell_3$Cell_16), 1)

piepercent18 <- round(100*Isoform_expression_12cell_3$Cell_18/sum(Isoform_expression_12cell_3$Cell_18), 1)

piepercent19 <- round(100*Isoform_expression_12cell_3$Cell_19/sum(Isoform_expression_12cell_3$Cell_19), 1)

piepercent20 <- round(100*Isoform_expression_12cell_3$Cell_20/sum(Isoform_expression_12cell_3$Cell_20), 1)

pie(Isoform_expression_12cell_3$Cell_02, col =ColorJYK4, labels = piepercent02, main = "Cell_01", cex = 1)

pie(Isoform_expression_12cell_3$Cell_03, col =ColorJYK4, labels = piepercent03, main = "Cell_02", cex = 1)

pie(Isoform_expression_12cell_3$Cell_05, col =ColorJYK4, labels = piepercent05, main = "Cell_03", cex = 1)

pie(Isoform_expression_12cell_3$Cell_09, col =ColorJYK4, labels = piepercent09, main = "Cell_04", cex = 1)

pie(Isoform_expression_12cell_3$Cell_19, col =ColorJYK4, labels = piepercent19, main = "Cell_05", cex = 1)

pie(Isoform_expression_12cell_3$Cell_20, col =ColorJYK4, labels = piepercent20, main = "Cell_06", cex = 1)

pie(Isoform_expression_12cell_3$Cell_10, col =ColorJYK4, labels = piepercent10, main = "Cell_07", cex = 1)

pie(Isoform_expression_12cell_3$Cell_11, col =ColorJYK4, labels = piepercent11, main = "Cell_08", cex = 1)

pie(Isoform_expression_12cell_3$Cell_13, col =ColorJYK4, labels = piepercent13, main = "Cell_09", cex = 1)

pie(Isoform_expression_12cell_3$Cell_14, col =ColorJYK4, labels = piepercent14, main = "Cell_10", cex = 1)

pie(Isoform_expression_12cell_3$Cell_18, col =ColorJYK4, labels = piepercent18, main = "Cell_11", cex = 1)

pie(Isoform_expression_12cell_3$Cell_16, col =ColorJYK4, labels = piepercent16, main = "Cell_12", cex = 1)
