# Supplementary figures and images for: Identification of a novel isoform of Slc26a4 by single-cell RNA-sequencing of pendrin-expressing cells in the cochlea
Source: Hum Genet. 2026 Jul 16;145(1):62. doi: 10.1007/s00439-026-02858-x (PMC13375699; doi:10.1007/s00439-026-02858-x)

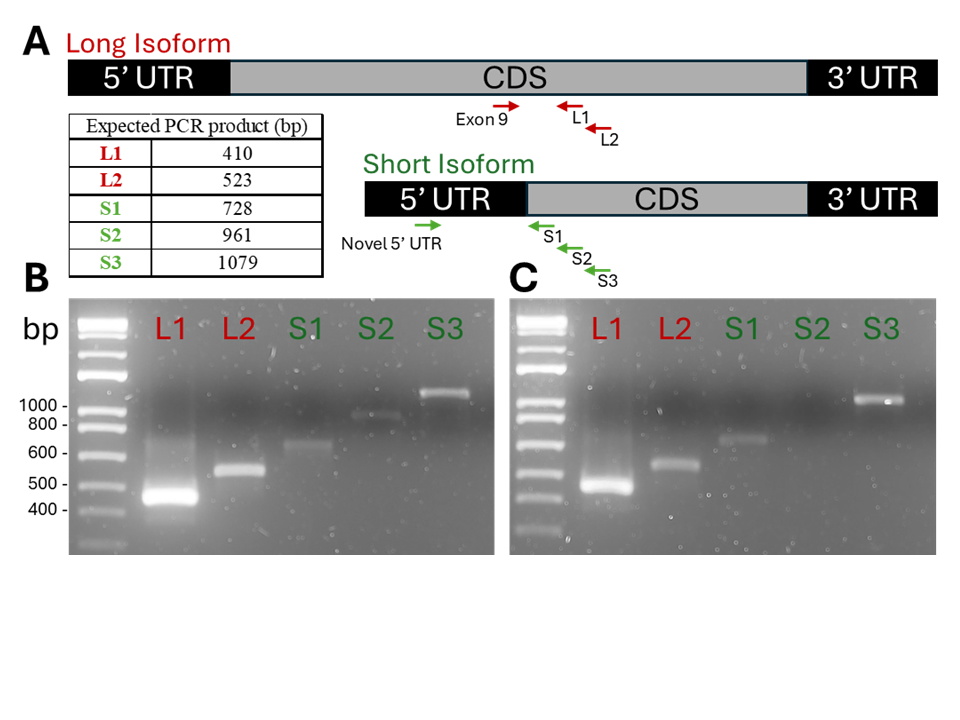

Supplement: Supplementary file 3 — Supplementary Material 3 [file 439_2026_2858_MOESM3_ESM.tif]

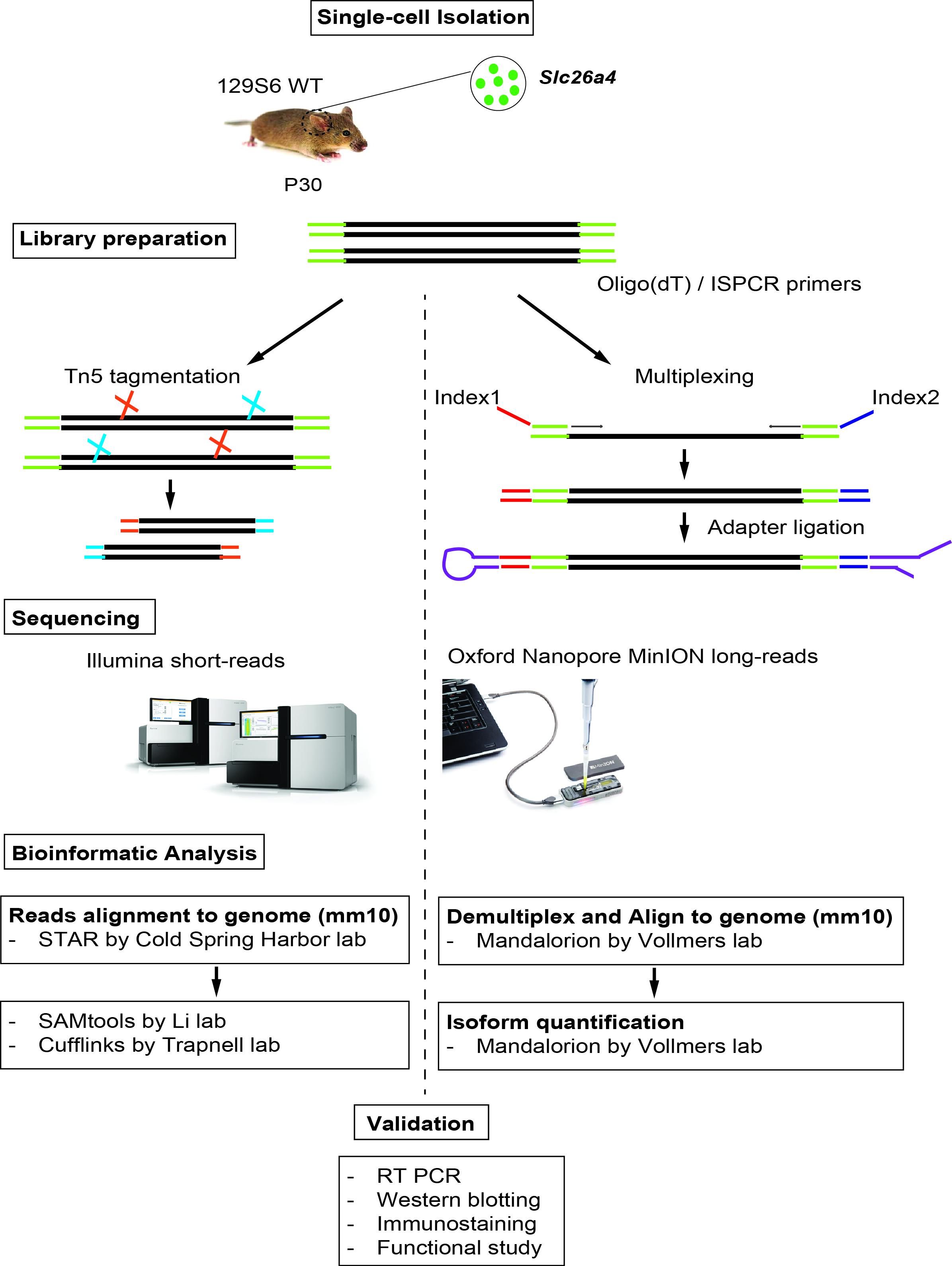

Supplement: Supplementary file 4 — Supplementary Material 4 [file 439_2026_2858_MOESM4_ESM.jpg]

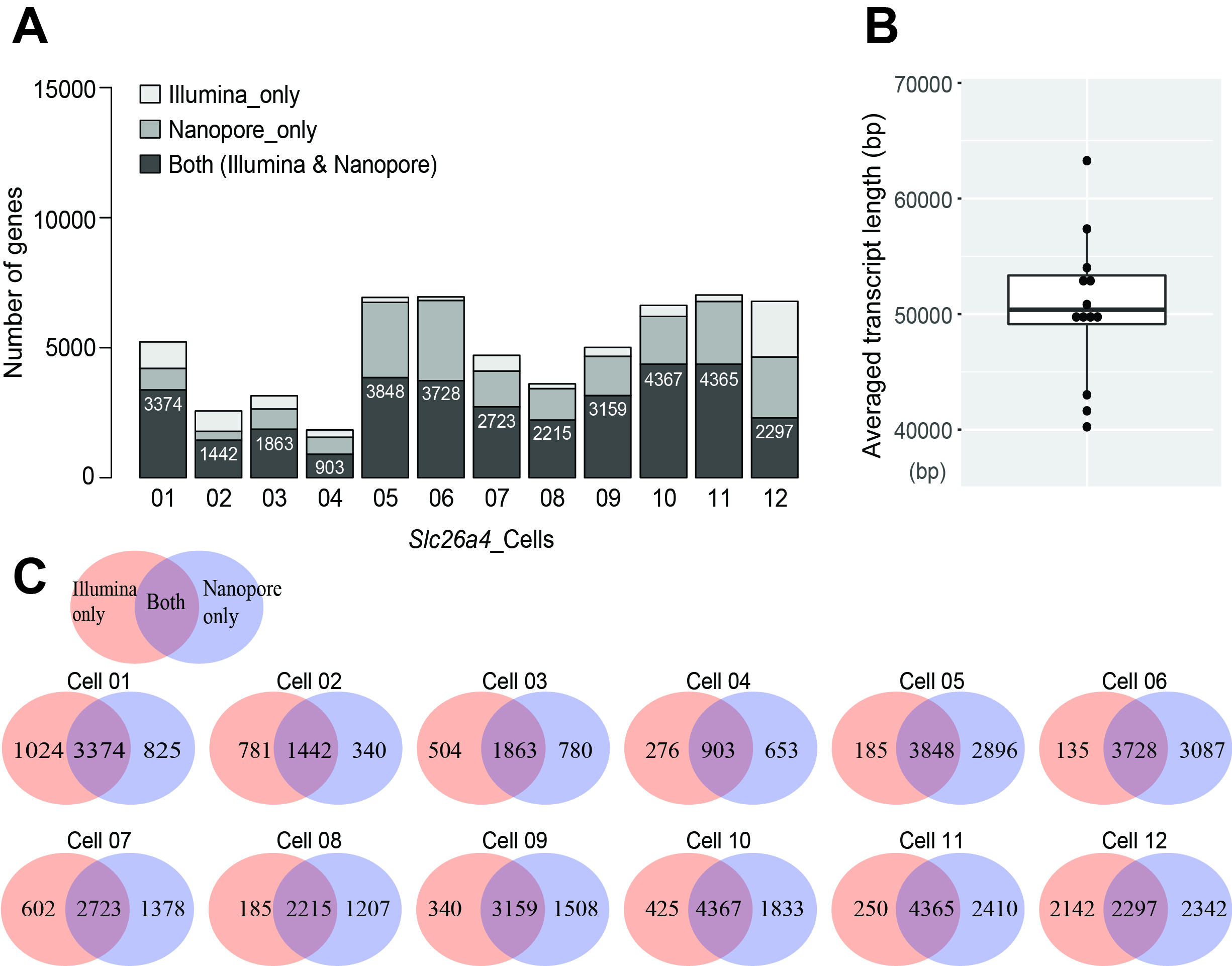

Supplement: Supplementary file 5 — Supplementary Material 5 [file 439_2026_2858_MOESM5_ESM.jpg]

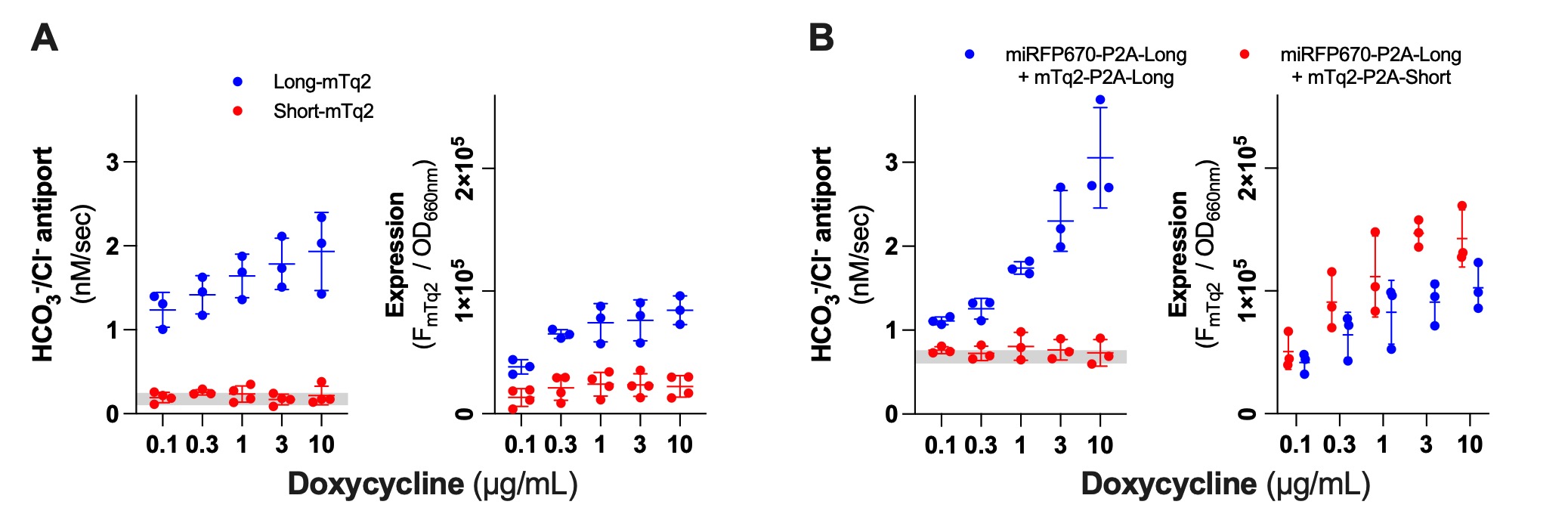

Supplement: Supplementary file 6 — Supplementary Material 6 [file 439_2026_2858_MOESM6_ESM.jpg]

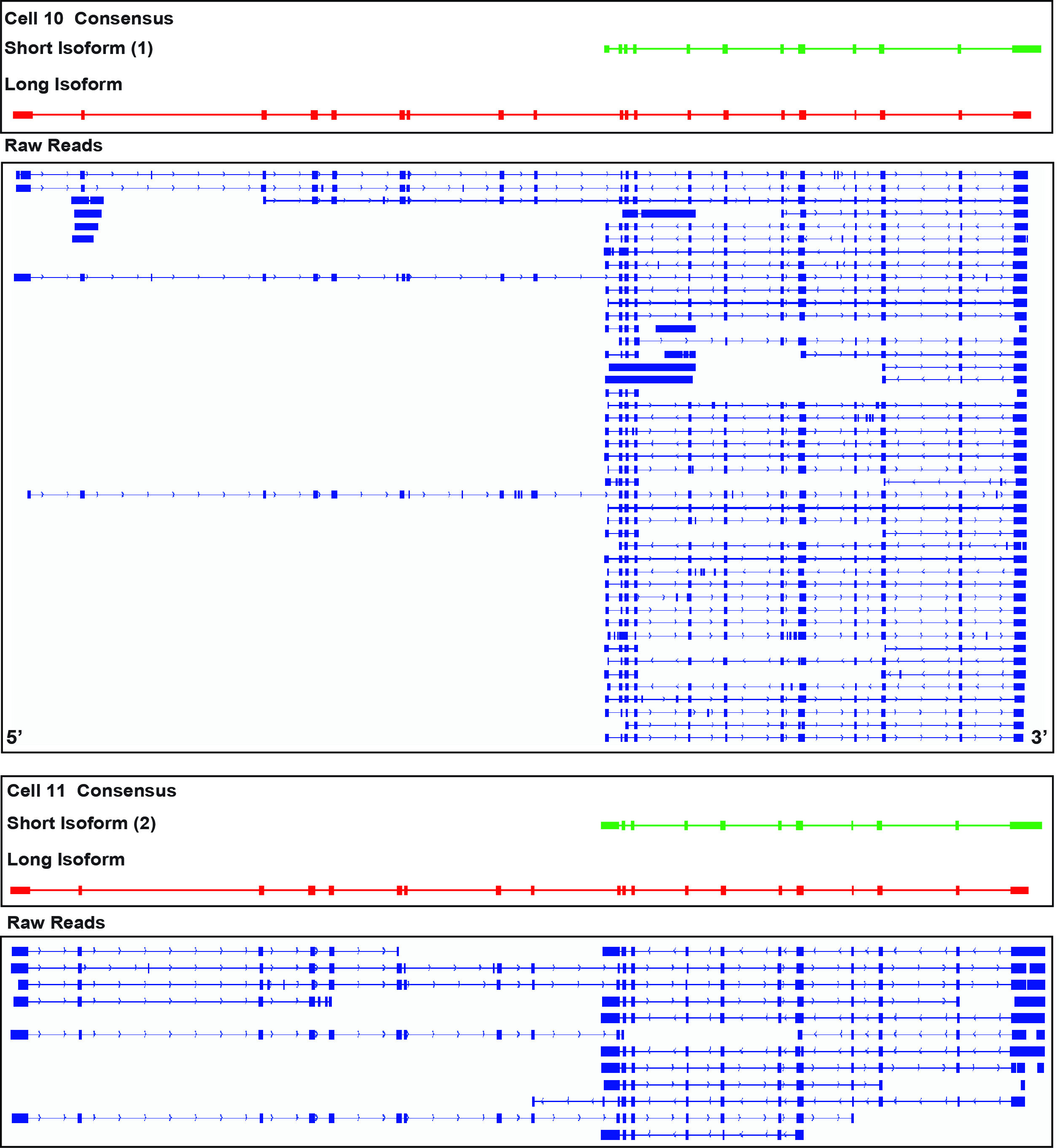

Supplement: Supplementary file 7 — Supplementary Material 7 [file 439_2026_2858_MOESM7_ESM.jpg]

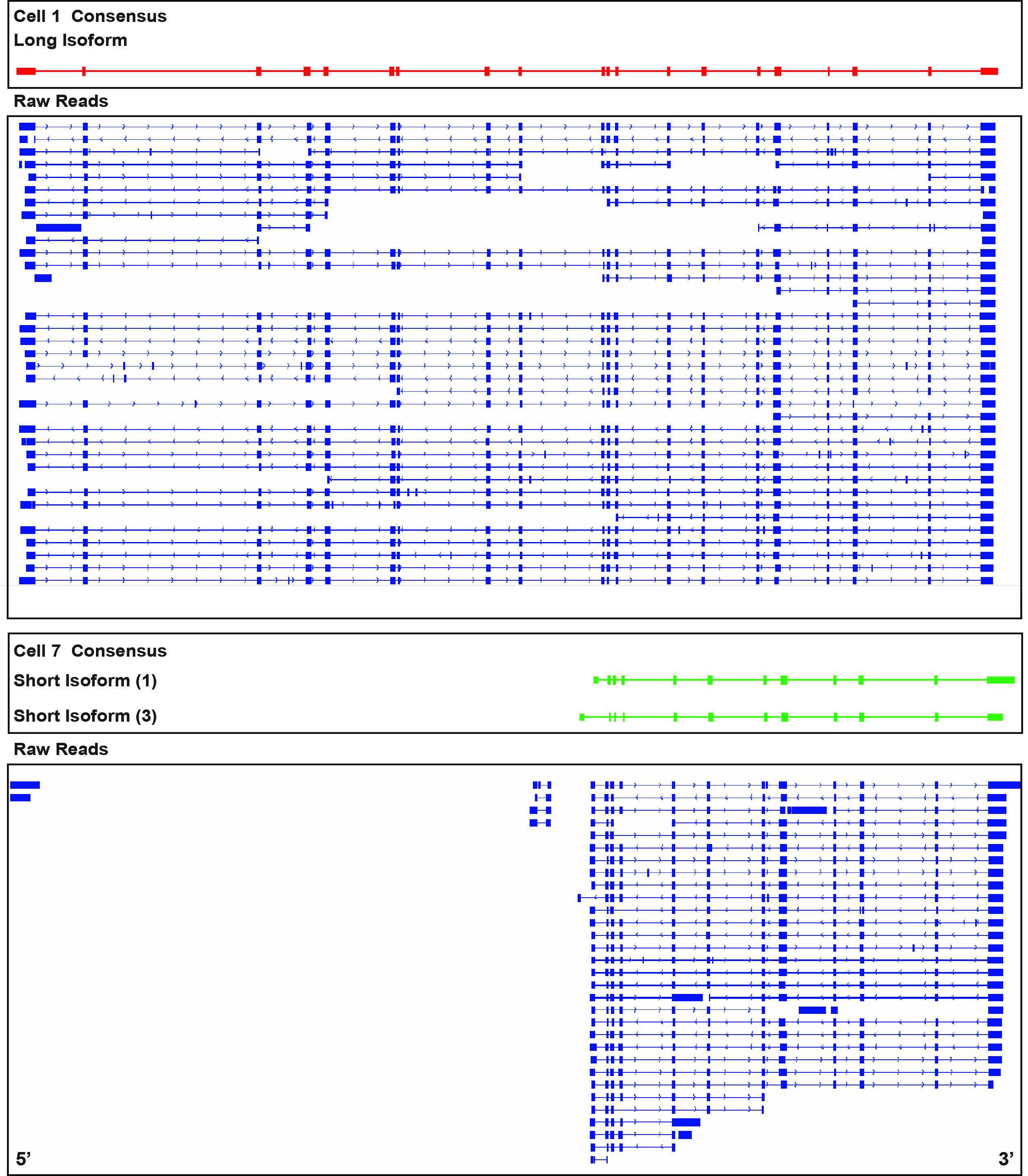

Supplement: Supplementary file 8 — Supplementary Material 8 [file 439_2026_2858_MOESM8_ESM.jpg]

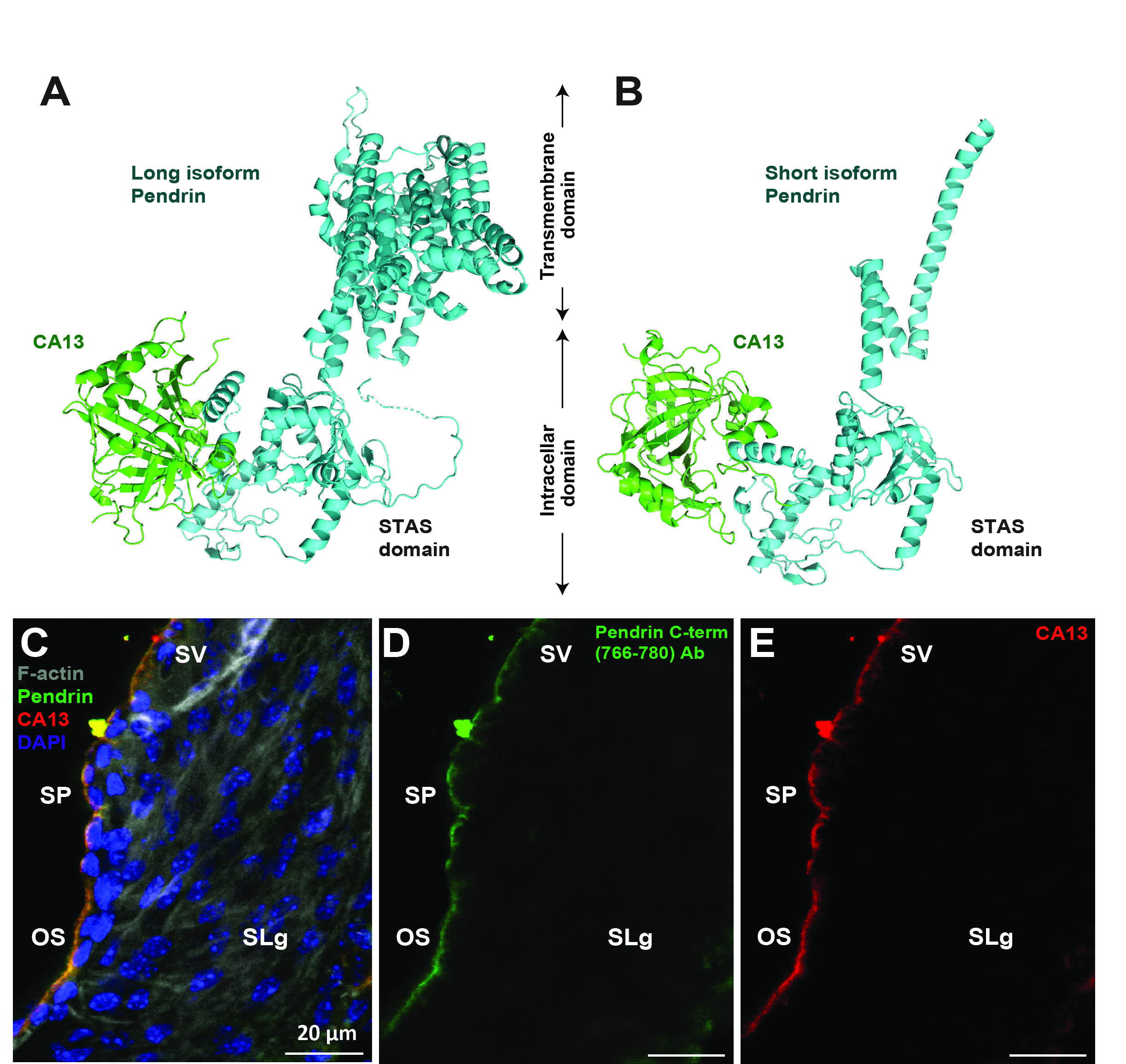

Supplement: Supplementary file 9 — Supplementary Material 9 [file 439_2026_2858_MOESM9_ESM.jpg]

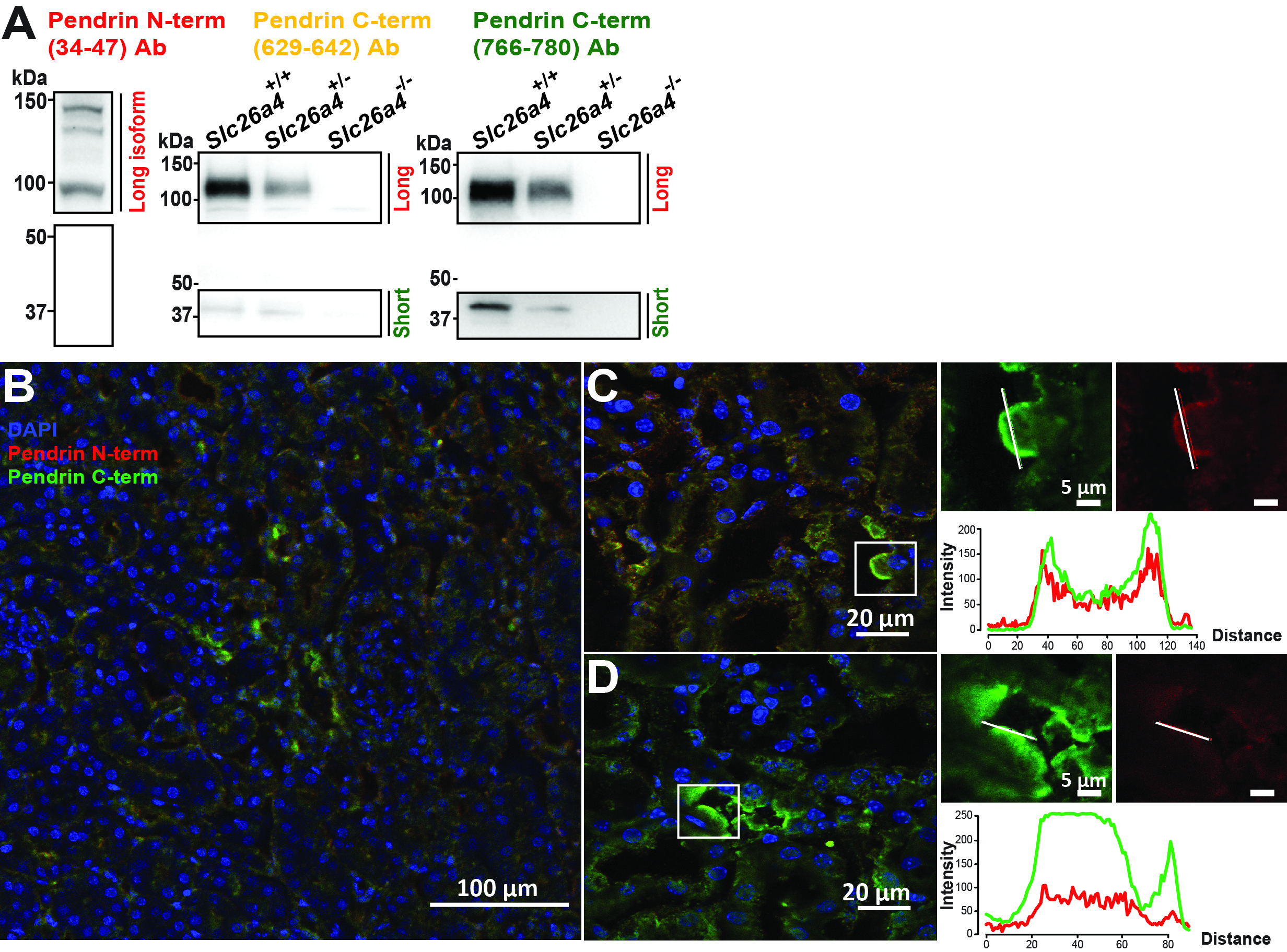

Supplement: Supplementary file 10 — Supplementary Material 10 [file 439_2026_2858_MOESM10_ESM.jpg]
